# Uncovering *Cis*-Regulatory Elements Important for A-to-I RNA Editing in Fusarium graminearum

**DOI:** 10.1128/mbio.01872-22

**Published:** 2022-09-14

**Authors:** Chanjing Feng, Xinyu Cao, Yanfei Du, Yitong Chen, Kaiyun Xin, Jingwen Zou, Qiaojun Jin, Jin-Rong Xu, Huiquan Liu

**Affiliations:** a State Key Laboratory of Crop Stress Biology for Arid Areas, College of Plant Protection, Northwest A&F University, Yangling, Shaanxi, China; b Department of Botany and Plant Pathology, Purdue University, West Lafayette, Indiana, USA; University of Georgia

**Keywords:** RNA editing, sexual reproduction, perithecia, sequence preference, secondary structure, *Fusarium graminearum*, *cis*-acting elements

## Abstract

Adenosine-to-inosine (A-to-I) RNA editing independent of adenosine deaminase acting on RNA (ADAR) enzymes was discovered in fungi recently, and shown to be crucial for sexual reproduction. However, the underlying mechanism for editing is unknown. Here, we combine genome-wide comparisons, proof-of-concept experiments, and machine learning to decipher *cis*-regulatory elements of A-to-I editing in Fusarium graminearum. We identified plenty of RNA primary sequences and secondary structural features that affect editing specificity and efficiency. Although hairpin loop structures contribute importantly to editing, unlike in animals, the primary sequences have more profound influences on editing than secondary structures. Nucleotide preferences at adjacent positions of editing sites are the most important features, especially preferences at the −1 position. Unexpectedly, besides the number of positions with preferred nucleotides, the combination of preferred nucleotides with depleted ones at different positions are also important for editing. Some *cis*-sequence features have distinct importance for editing specificity and efficiency. Machine learning models built from diverse sequence and secondary structural features can accurately predict genome-wide editing sites but not editing levels, indicating that the *cis*-regulatory principle of editing efficiency is more complex than that of editing specificity. Nevertheless, our model interpretation provides insights into the quantitative contribution of each feature to the prediction of both editing sites and levels. We found that efficient editing of FG3G34330 transcripts depended on the full-length RNA molecule, suggesting that additional RNA structural elements may also contribute to editing efficiency. Our work uncovers multidimensional *cis*-regulatory elements important for A-to-I RNA editing in F. graminearum, helping to elucidate the fungal editing mechanism.

## INTRODUCTION

RNA editing is an epigenetic phenomenon that alters the RNA sequence so that it differs from the genomic DNA sequence. Types of RNA editing include insertion, deletion, and base modification of nucleotides (1). The most common type of RNA editing is adenosine-to-inosine (A-to-I) editing (2). It is highly prevalent across the animal kingdom (3, 4). Especially, in humans and octopuses, millions of A-to-I editing sites have been identified (5, 6). Since cellular machinery recognizes I as guanosine (G), A-to-I editing has the same consequence as A-to-G mutation in RNA. Editing in the coding region of RNA molecule may result in non-synonymous substitutions, leading to protein recoding. Despite the capacity for protein recoding, the vast majority of A-to-I editing sites in animals occur in non-coding regions associated with repetitive elements and the recoding editing events are relatively rare (3, 7).

Although it has been studied for over 30 years in animals, A-to-I RNA editing was discovered in fungi only recently (8). To date, genome-wide A-to-I RNA editing has been reported to occur specifically during sexual reproduction in several filamentous ascomycetes, including Fusarium graminearum (8), Neurospora crassa (9), and Pyronema confluens (10). Unlike in animals, the majority of A-to-I editing sites are recoding editing sites and generally adaptive in fungi (9, 11, 12).

In animals, A-to-I RNA editing is mediated by members of the adenosine deaminase acting on RNA (ADAR) family (13). The ADAR family members share common functional domains with a catalytic deaminase domain at the C-terminal region and one to three repeats of double-stranded RNA (dsRNA)-binding domain (dsRBD) at the N-terminal region. ADAR enzymes only recognize adenosines within dsRNA. Repetitive elements can readily hybridize to form dsRNA, which is the preferred target of the ADAR enzymes. Each metazoan genome encodes at least one member of the ADAR family. Since the ADAR family appear to be a metazoan innovation, there is no ADAR ortholog in fungi. The editing machinery in fungi remains to be determined.

In a cell, not all adenosines in an RNA substrate and not every transcript at a given site are edited. The percentage of edited transcripts over total transcripts at a given site is defined as editing level (or efficiency), which can vary from very low to nearly 100%. The underlying mechanisms determining which sites within the dsRNA to be edited (editing specificity) and to what levels (editing efficiency) by ADAR enzymes have been extensively investigated in animals. Multiple RNA sequence and structure features (*cis*-acting regulatory elements) have been proposed to regulate ADAR editing, including nucleotides opposing the target adenosine, 5′ and 3′ nearest-neighboring nucleotides next to the editing site, local secondary structure, length and stability of dsRNA structure, and tertiary structure of the RNA substrate (14, 15). The ADAR enzymes do not recognize a strict consensus sequence but have a weak nucleotide preference adjacent to the editing site, including a bias against a G at the −1 position and a slight enrichment for G or A at the +1 position (3). In fungi, however, a strong nucleotide preference surrounding the editing sites has been observed (8, 9, 12). The A-to-I editing sites in fungi were significantly enriched in hairpin loop structures rather than dsRNA structures predicted from the immediate vicinity of the edited sites (8, 9, 12). Nevertheless, whether, and to what extent, these observed sequence and secondary structure preferences contribute to editing specificity and efficiency is unclear in fungi.

F. graminearum is the predominant causal agent of Fusarium head blight (FHB) (16), one of the most devastating diseases on cereal crops worldwide. It is a haploid homothallic ascomycete and produces abundant perithecia by sexual reproduction in both field and laboratory conditions (17). Ascospores are forcibly discharged from mature perithecia and dispersed by wind as the primary inoculum of FHB (18, 19). F. graminearum is the first fungus in which the A-to-I RNA editing was discovered (8). Strand-specific RNA-seq analysis identified 26,056 A-to-I editing sites in matured perithecia. A-to-I RNA editing of 3 genes *PUK1*, *FgAMA1*, and *AMD1* has been demonstrated to be important for ascospore formation and discharge in F. graminearum (8, 20, 21). A-to-I RNA editing was also shown to increase the proteomic diversity that confers an adaptive advantage in both F. graminearum and N. crassa (9, 11). Therefore, it has been proposed that fungi-specific RNA editing machinery is an ideal drug target to control the fungal plant pathogens that use ascospores as the primary inoculum (22, 23).

In this study, we dissected the *cis*-regulatory sequence and structural elements that affect A-to-I RNA editing in F. graminearum by genome-wide comparisons. As a proof-of-concept, we chose 4 representative editing sites to evaluate the importance of different RNA sequence and structure features for their editing levels using a site-specific mutagenesis approach at the native locus. Based on the identified *cis*-regulatory features, we also used supervised machine learning to build predictive models of RNA editing sites and levels and quantified the contributions of all features to the RNA editing predictions. Our results provide insights into the complex *cis*-regulation of A-to-I RNA editing and help to elucidate the editing mechanism in fungi.

## RESULTS

### Expanding the landscape of A-to-I RNA editing sites in F. graminearum.

To detect more A-to-I RNA editing sites in F. graminearum, we generated over 17 Gb strand-specific RNA-seq data from 5 biological replicates of perithecia collected at 6 days post-fertilization (dpf) (Table S1). In combination with our previous RNA-seq data from 2 biological replicates of perithecia (8), we identified a total of 40,235 bona fide A-to-I editing sites with a low false discovery rate of 0.18% (Table S2). The number of detected A-to-I editing sites was comparable with that (40,677) in N. crassa (9) but over a 1.5-fold increase in comparison with the previous report (26,056) in F. graminearum (8). The editing level of identified A-to-I sites in F. graminearum varied from 3% (the cutoff value used) to 100%, with a median value of 12%. More than 80% of sites had editing levels <30%, whereas less than 4% had editing levels >60%. The fraction of sites with low editing levels was increased relative to the previous report in F. graminearum (8), suggesting that a large number of editing sites with low editing levels can only be detected under a high read coverage by the combination of multiple samples.

10.1128/mbio.01872-22.6 TABLE S1 RNA-seq and DNA-seq data used. Download Table S1, DOCX file, 0.01 MB. Copyright © 2022 Feng et al. 2022 Feng et al. https://creativecommons.org/licenses/by/4.0/ This content is distributed under the terms of the Creative Commons Attribution 4.0 International license.

10.1128/mbio.01872-22.7 TABLE S2 The number of different RNA variant sites detected in Fusarium graminearum. Download Table S2, DOCX file, 0.01 MB. Copyright © 2022 Feng et al. 2022 Feng et al. https://creativecommons.org/licenses/by/4.0/ This content is distributed under the terms of the Creative Commons Attribution 4.0 International license.

### Multidimensional RNA sequence features important for A-to-I editing.

**FIG 1:**
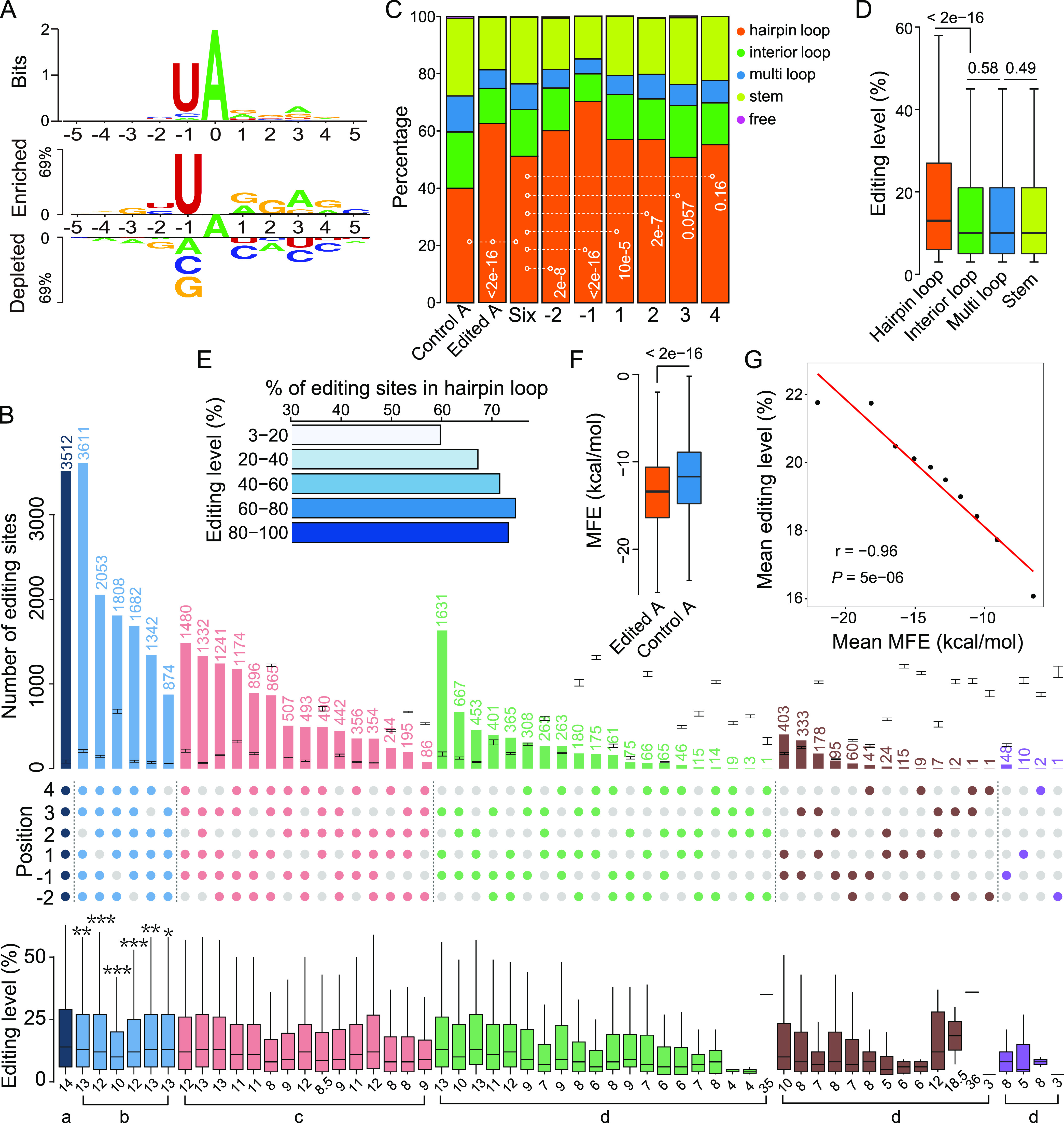
*Cis*-regulatory elements of A-to-I RNA editing in F. graminearum. (A) Nucleotide preferences in the flanking sequences of the editing sites. The height of letters in WebLogo (Upper) depicts the information content of the position in bits. Two Sample Logo (Lower) showing the enriched and depleted nucleotides surrounding the editing sites in comparison with negative control (an equal number of A sites randomly selected from edited transcript sequences) (*t* test, *P < *0.0001). (B) The number of editing sites and their editing levels in each group categorized by the combination of preferred and depleted nucleotides at −2 to +4 positions. Gray dots represent depleted nucleotides. Other colored dots represent groups with different numbers of preferred nucleotides. The number of editing sites in each group is indicated above the bar and the median editing level is indicated under the boxplot. Means and standard deviations (error bars on the bar chart) for the number of editing sites in each group were calculated with 3 replicates of an equal number of randomly selected A sites. Different letters indicate significant differences among groups based on ANOVA analysis followed by Scheffé’s test (*P < *0.05). Asterisks indicate significant differences relative to the group with preferred nucleotides at all six positions (*, *P < *0.05; **, *P < *0.01; ***, *P < *0.001; *t* test). (C) Stacked columns showing fractions of different types of RNA secondary structure elements predicted based on 30-nt upstream and 30-nt downstream sequences of the control A sites, all editing sites, editing sites with preferred nucleotides at all six positions (Six), and editing sites with depleted nucleotides at one position and preferred nucleotides at the other 5 positions (−2 to 4). For control, an equal number of A sites with similar nucleotide preference at the −2 to +4 positions were randomly selected from edited transcript sequences. *P*-value is from the χ^2^ test. (D) Boxplots comparing the editing level of the editing sites located in different types of RNA secondary structure elements. *P*-values are from two-tailed Wilcoxon rank-sum tests. (E) Percentage of editing sites with marked editing levels located in hairpin loops. (F) Boxplots comparing the minimum free energy (MFE) of predicted hairpin loops for edited and control A sites. *P*-value is from the two-tailed Wilcoxon rank-sum test. (G) Correlation between the mean editing level and MFE. Each dot represents 10% of editing sites located in hairpin loops. The Pearson correlation coefficient and *P-value* are indicated.

WebLogo analysis of all the identified A-to-I editing sites in F. graminearum showed a conserved sequence motif surrounding the editing sites from −2 to +4 positions (Fig. 1A). In comparison with randomly selected A sites (*t* test, *P < *0.0001), the −1 position of editing sites was strongly enriched for uridine (U) but depleted for the other 3 nucleotides. Whereas the −2 position was slightly enriched for cytidine (C)/U but depleted for A/G, the +1, +3, and +4 positions were slightly enriched for A/G but depleted for C/U (Fig. 1A). The +2 position was slightly enriched for G but depleted for C/A. Therefore, consistent with previous observations (8), the A-to-I RNA editing in F. graminearum has strong primary sequence preferences surrounding the editing sites.

To investigate the influence of nucleotide preferences on RNA editing, we divided all the editing sites into different groups according to the combinations of preferred (enriched) and depleted nucleotides at −2 to +4 positions (Fig. 1B). The group with preferred nucleotides at all 6 positions contained 3,512 (8.7%) editing sites while that with depleted nucleotides at all 6 positions had only 2. In comparison with the group with preferred nucleotides at all 6 positions, the groups with depleted nucleotides at one position and preferred nucleotides at the other 5 positions had fewer editing sites, except the group with depleted nucleotides at the +2 position. The group with depleted nucleotides at the +2 position and preferred nucleotides at the other 5 positions had more editing sites but the expected number of editable sites (random sampling) was also higher for this group. These results suggest that except for the +2 position preferred nucleotides at each of the other 5 positions contribute importantly to RNA editing specificity. When depleted nucleotides occurred at the −1 position, all the groups had editing sites far fewer than expected, except the group with preferred nucleotides at all other 5 positions. These results suggest that the -1 position is the most crucial one for editing specificity.

When preferred nucleotides occurred at more positions, the fraction of groups with editing sites more than expected was increased (Fig. 1B), suggesting that the number of positions with preferred nucleotides affects RNA editing specificity in F. graminearum. It should be noted that the group with preferred nucleotides at more positions were not necessary to have more editing sites. For example, the group with preferred nucleotides at −1, +1, and +3 positions and depleted nucleotides at the other 3 positions contained a larger number of editing sites than that of many groups with preferred nucleotides beyond the −1, +1, and +3 positions (Fig. 1B). Additionally, among the groups with preferred or depleted nucleotides at the same number of positions, the number of editing sites varied extensively (Fig. 1B). These observations indicate that the combination of preferred nucleotides with depleted ones at different positions is also important for RNA editing specificity in F. graminearum.

In comparison with the group with preferred nucleotides at all 6 positions, the groups with depleted nucleotides at one position and preferred nucleotides at the other 5 positions had lower median editing levels, especially the group with depleted nucleotides at the −1 position. These results suggest that the nucleotide preferences at each of the 6 positions play important roles in RNA editing efficiency and the −1 position has a more profound influence. Like editing sites, the editing levels tended to be higher for the groups with preferred nucleotides at more positions and depleted nucleotides at fewer positions. The median editing levels also varied among the groups with different combinations of preferred or depleted nucleotides at different positions. Therefore, the number and combination mode of different positions of preferred and depleted nucleotides also affect RNA editing efficiency in F. graminearum.

### RNA secondary structure features important for A-to-I editing.

Next, we predicted the secondary structures of RNA sequences surrounding the editing sites (30 nt upstream and 30 nt downstream). Consistent with previous observations (8), over 62% of editing sites resided in the predicted hairpin loops, which is significantly higher than that of the control A sites (*χ^2^* test, *P < *2e-16) (Fig. 1C). A similar conclusion was also obtained when the secondary structures were predicted using the full-length RNA sequences, although the fraction of predicted hairpin loops was decreased for both edited and control A sites (Fig. S1). In addition, the editing sites in hairpin loops had a higher median editing level compared with those in other RNA secondary structure elements (Fig. 1D), and the editing sites with higher editing levels were more likely located in hairpin loops (Fig. 1E). These results suggest that RNA secondary structures have important roles in the specificity and efficiency of RNA editing.

10.1128/mbio.01872-22.1 FIG S1 Stacked columns showing the percentage of edited sites with marked editing levels and control sites in the 5 types of RNA secondary structure elements predicated from full-length RNA sequences. The full-length RNA sequence of each gene was obtained from FgBase (http://fgbase.wheatscab.com/). Download FIG S1, PDF file, 0.1 MB. Copyright © 2022 Feng et al. 2022 Feng et al. https://creativecommons.org/licenses/by/4.0/ This content is distributed under the terms of the Creative Commons Attribution 4.0 International license.

Analyzing the minimum free energy (MFE) of predicated RNA secondary structures revealed that the hairpin loops with editing sites had a lower MFE value (more stable) compared to those with control A sites (Fig. 1F), and the editing level inversely correlated with the MFE value of hairpin loops (Fig. 1G). These results suggest that the stability of hairpin loops is also important for RNA editing. Similar results were obtained for the A-to-I RNA editing sites in N. crassa (Fig. S2), suggesting that there are conserved *cis*-acting elements regulating A-to-I RNA editing in fungi.

10.1128/mbio.01872-22.2 FIG S2 *Cis*-regulatory elements of A-to-I RNA editing in Neurospora crassa. (A) Boxplots comparing the editing levels of editing sites with enriched and depleted nucleotides at -2 to +3 positions. (B) Percentage of editing sites with marked editing levels located in hairpin loops. (C) Boxplots comparing the editing level of the editing sites located in hairpin loops and other types of RNA secondary structure elements. (D) Boxplots comparing the minimum free energy (MFE) of predicted hairpin loops for edited and control A sites. (E) Correlation between the mean editing level and the mean MFE. Each dot represents 10% of editing sites located in hairpin loops. The Pearson correlation coefficient and *P*-value are indicated. *P*-values in (A), (C), and (D) are from two-tailed Wilcoxon rank-sum tests. Download FIG S2, PDF file, 1.5 MB. Copyright © 2022 Feng et al. 2022 Feng et al. https://creativecommons.org/licenses/by/4.0/ This content is distributed under the terms of the Creative Commons Attribution 4.0 International license.

Intriguingly, in comparison with all the editing sites in general, the editing sites with preferred nucleotides at all 6 positions had lower fractions of sites located in hairpin loops (Fig. 1C). Moreover, relative to the group with preferred nucleotides at all 6 positions, the fraction of editing sites located in hairpin loops were increased in the groups with depleted nucleotides at −2, −1, +1, or +2 position and preferred nucleotides at the other 5 positions, especially the group with depleted nucleotides at −1 position. These results indicate that the hairpin loop structure is more important for editing the A’s with less preferred neighboring sequences. Although these groups had higher fractions of sites located in hairpin loops, their editing levels were significantly lower (*t* test, *P < *0.05), implying that the influence of secondary structure on RNA editing is not as critical as primary sequences.

### Selection of FG3G34330 for characterizing *cis*-acting elements of A-to-I editing.

**FIG 2:**
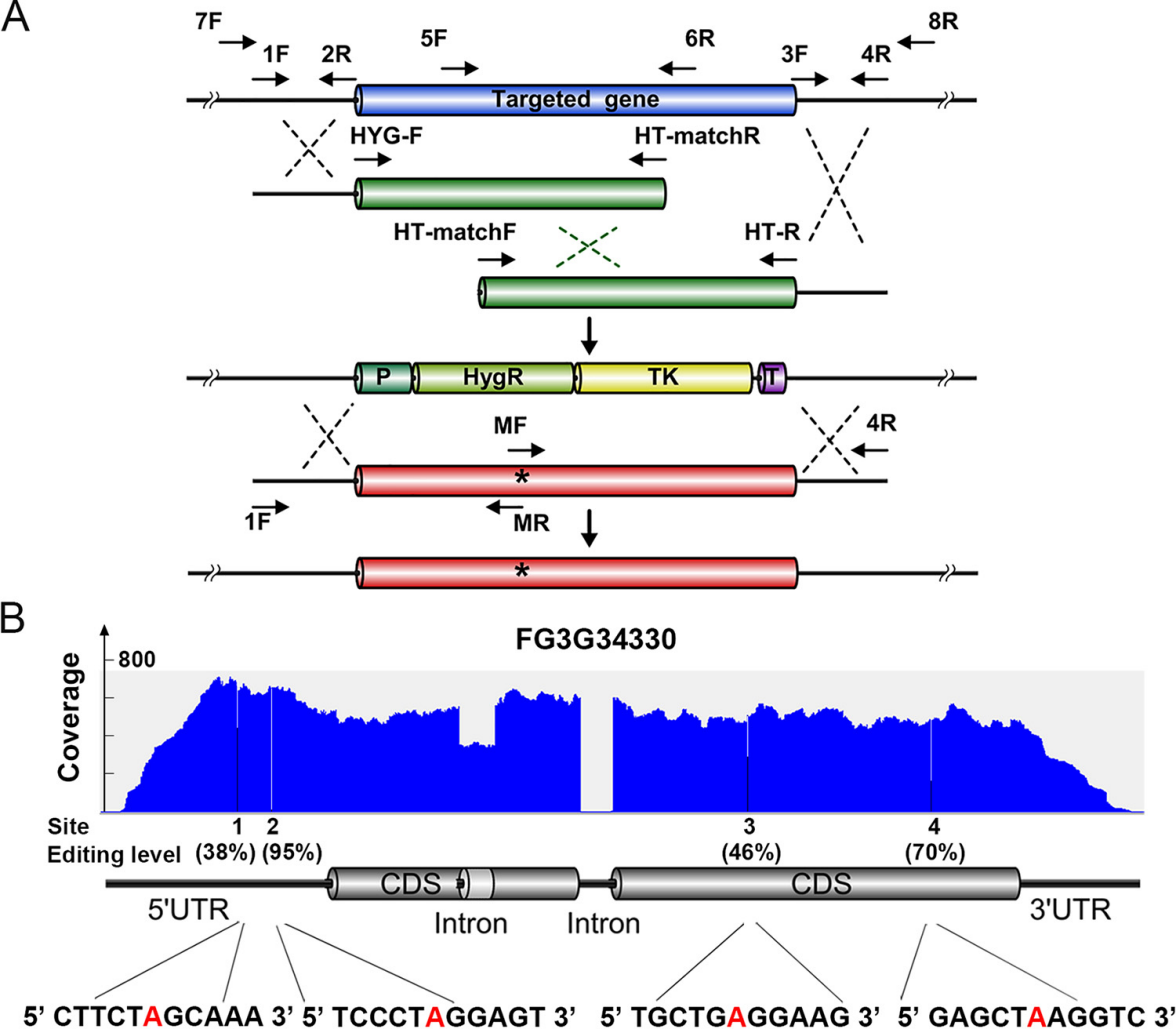
Allelic exchange strategy and candidate gene used in this study. (A) Graphical representation of the gene deletion and allelic exchange strategy used in this study. The selectable marker cassette expresses a translational fusion of the hygromycin phosphotransferase (HygR) with the thymidine kinase (TK) that confers resistance to the antibiotic hygromycin and sensitivity to nucleoside analog 5-fluoro-2’-deoxyuridine (Floxuridine). Allelic fragments with desired mutations (marked with *) were generated by overlapping PCR. The location of PCR primers (F, forward and R, reverse) are indicated. P, promoter; T, terminator. (B) Gene structure, RNA-seq read coverage, and editing sites of FG3G34330 used for testing the effects of *cis*-acting elements on A-to-I RNA editing. The position, editing level, and neighboring sequences of the four editing A sites (in red) are shown. CDS, coding sequences; UTR, untranslated region.

To verify the effect of neighboring nucleotides and secondary structures on RNA editing in F. graminearum, we adopted a recyclable marker module to perform gene deletion and allelic exchange at the native genomic locus (Fig. 2A). We selected a gene FG3G34330 that encodes a hypothetical protein without known conserved domains. It was highly expressed in perithecia but low expressed in other stages (Fig. S3). The transcript of FG3G34330 had 4 editing sites with variable editing levels from 38% to 95% (Fig. 2B). All 4 editing sites (numbered 1 to 4 from 5′- to 3′-end) had preferred nucleotides at −2 to +4 positions, except the +2 position of editing site 1 and the −1 position of editing site 3, which had depleted nucleotides C and G, respectively. Consistently, the editing levels of sites 1 and 3 were lower than those of sites 2 and 4.

10.1128/mbio.01872-22.3 FIG S3 RNA-seq expression of FG3G34330 gene in different samples. The Transcripts Per Kilobase of exon model per Million mapped reads (TPM) expression value was obtained from FgBase (http://fgbase.wheatscab.com/). Coni, conidia collected from 5-day-old CMC cultures; Ger12h and Hyp24h, germlings and vegetative hyphae collected from conidia incubated for 12 and 48 h in liquid YEPD cultures; Inf3d, inoculated spikelets of flowering wheat heads collected 3 days after inoculation; TBI3d, DON-producing hyphae collected from 3-d-old liquid trichothecene biosynthesis induction (TBI) cultures; Sex3d and Sex8d, perithecia harvested from carrot agar plates at 3- or 8-days postfertilization. Download FIG S3, PDF file, 0.04 MB. Copyright © 2022 Feng et al. 2022 Feng et al. https://creativecommons.org/licenses/by/4.0/ This content is distributed under the terms of the Creative Commons Attribution 4.0 International license.

Deletion of FG3G34330 did not cause obvious phenotypic changes in vegetative growth and sexual development (Fig. S4), indicating that site-directed mutagenesis of this gene would not impact the normal development of F. graminearum and thereby our quantification of editing levels. Therefore, FG3G34330 and its editing sites are good candidates for investigating *cis*-acting elements of A-to-I RNA editing in F. graminearum.

10.1128/mbio.01872-22.4 FIG S4 Morphology of colonies, perithecia, asci, and ascospores generated by wild type (WT) and FG3G34330 deletion mutant (*ΔFG3G34330*). Bar = 20 μm. Download FIG S4, PDF file, 0.4 MB. Copyright © 2022 Feng et al. 2022 Feng et al. https://creativecommons.org/licenses/by/4.0/ This content is distributed under the terms of the Creative Commons Attribution 4.0 International license.

### Preferred U at −1 position is crucial for editing FG3G34330 transcripts.

**FIG 3:**
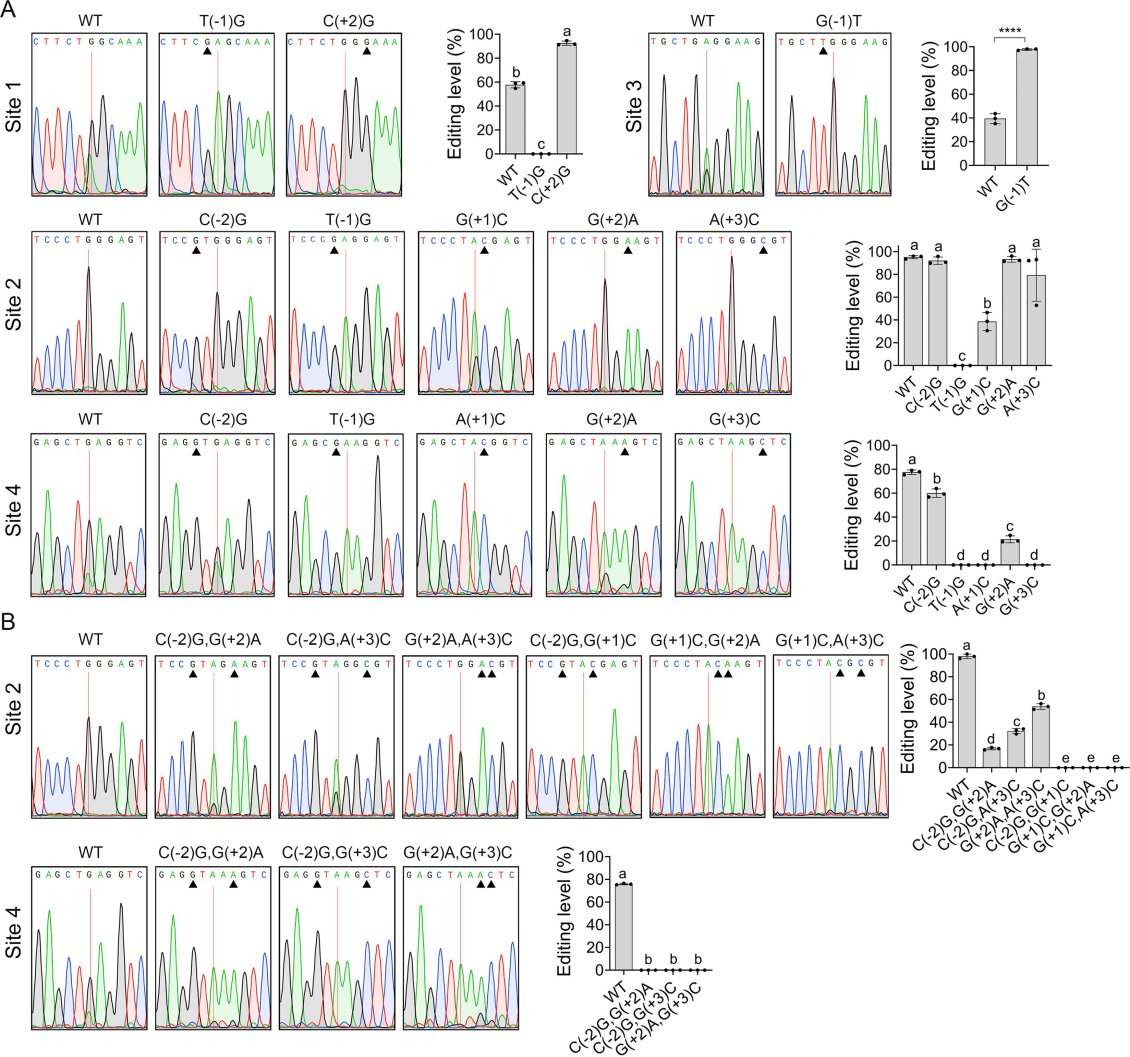
Effects of neighboring nucleotides on RNA editing of FG3G34330. (A) Effects of individual neighboring nucleotides on RNA editing of sites 1 to 4. (B) The combined effect of neighboring nucleotides on RNA editing of sites 2 and 4. Sequencing traces for flanking sequences of the editing sites of FG3G34330 amplified from RNA isolated from perithecia of wild type (WT) and mutants labeled by pre- and post-mutated bases and mutation positions relative to editing sites. Black triangles mark the mutation sites in each mutant. Red lines mark the editing sites that have mixed peaks of A and G in sequencing traces of WT although only the dominating peak is shown in the sequences on the top. Means and standard deviations of the editing levels were estimated from three biological replicates (*n* = 3). Different letters indicate significant differences based on ANOVA analysis followed by Duncan’s multiple range test (*P < *0.05). ****Indicates significant differences (*P < *0.0001) based on the *t* test.

To verify the effects of nucleotide preferences on editing, we first changed the preferred T(U) at the −1 position of editing sites 1, 2, and 4 into the depleted G at the native genomic locus of FG3G34330. Reverse-transcription (RT)-PCR amplification was performed with 6-dpf perithecia of obtained mutant and wild type (Table S3). Sanger sequencing of the PCR products showed that 2 peaks (A and G) occurred at the editing sites in the sequencing traces of wild type (Fig. 3). The relative proportion of G peak at the editing sites was quantified to determine their editing level. Consistent with its greatest enrichment, replacing the preferred U with the depleted G abolished the editing of sites 1, 2, and 4 (Fig. 3A). In addition, we changed the depleted G at the −1 position of editing site 3 into preferred T (U). In comparison with the wild type, the average editing level was dramatically increased from 40% to 98% in the resulting G(−1) to T mutant (Fig. 3A). These results demonstrate that the preferred U at the −1 position plays a crucial role in the A-to-I RNA editing in F. graminearum.

10.1128/mbio.01872-22.8 TABLE S3 Summary of strains used. Download Table S3, DOCX file, 0.02 MB. Copyright © 2022 Feng et al. 2022 Feng et al. https://creativecommons.org/licenses/by/4.0/ This content is distributed under the terms of the Creative Commons Attribution 4.0 International license.

### The importance of nucleotide preferences at other positions varies among different editing sites of FG3G34330.

To verify the effects of nucleotide preferences at other positions on the editing, we individually changed the preferred nucleotides of editing sites 2 and 4 of FG3G34330 at −2 and +1 to +3 positions into the depleted nucleotides. At the +1 position, replacing preferred G with depleted C dramatically reduced the average editing level of site 2 from 95% to 39% while replacing preferred A with depleted C abolished the editing of site 4 (Fig. 3A). Replacing the preferred nucleotides at −2, +2, or +3 positions with depleted nucleotides did not significantly affect the editing level of site 2 (analysis of variance [ANOVA], *P > *0.05), but markedly reduced the editing level of site 4 (Fig. 3A). Particularly, the editing of site 4 was completely disrupted in the G(+3)C mutant. In addition, we changed the depleted C at the +2 position of editing site 1 into preferred G. The average editing level was increased from 58% to 93% in the C(+2)G mutant relative to the wild type. Therefore, the influences of nucleotide preferences on editing at other positions vary among different editing sites and may be context-dependent.

### Combined effects of neighboring nucleotides on the editing of FG3G34330 transcripts.

Because individually replaced the preferred nucleotides at −2, +2, and +3 positions with depleted nucleotides did not significantly affect the editing level of site 2, we then examined whether they have combined effects on editing. Interestingly, although individual mutation at the −2, +2, and +3 positions did not affect editing levels, the editing level of site 2 was significantly reduced when they were mutated into depleted nucleotides in pairs (ANOVA, *P < *0.05) (Fig. 3B). Moreover, the editing of site 2 was abolished when its +1 position was mutated simultaneously with the −2, +2, or +3 positions in pairs from preferred to depleted nucleotides. Double mutations of the −2 and +2 positions also disrupted the editing of site 4 (Fig. 3B). Altogether, these results suggest that the neighboring nucleotides have large, combined effects on the editing. Noting that the effects of mutations at different pairs of 2, +2, and +3 positions were different, it was confirmed that the combined effects differ among combinations of preferred and depleted nucleotides at different positions.

### RNA secondary structures affect the editing efficiency of FG3G34330 transcripts.

**FIG 4:**
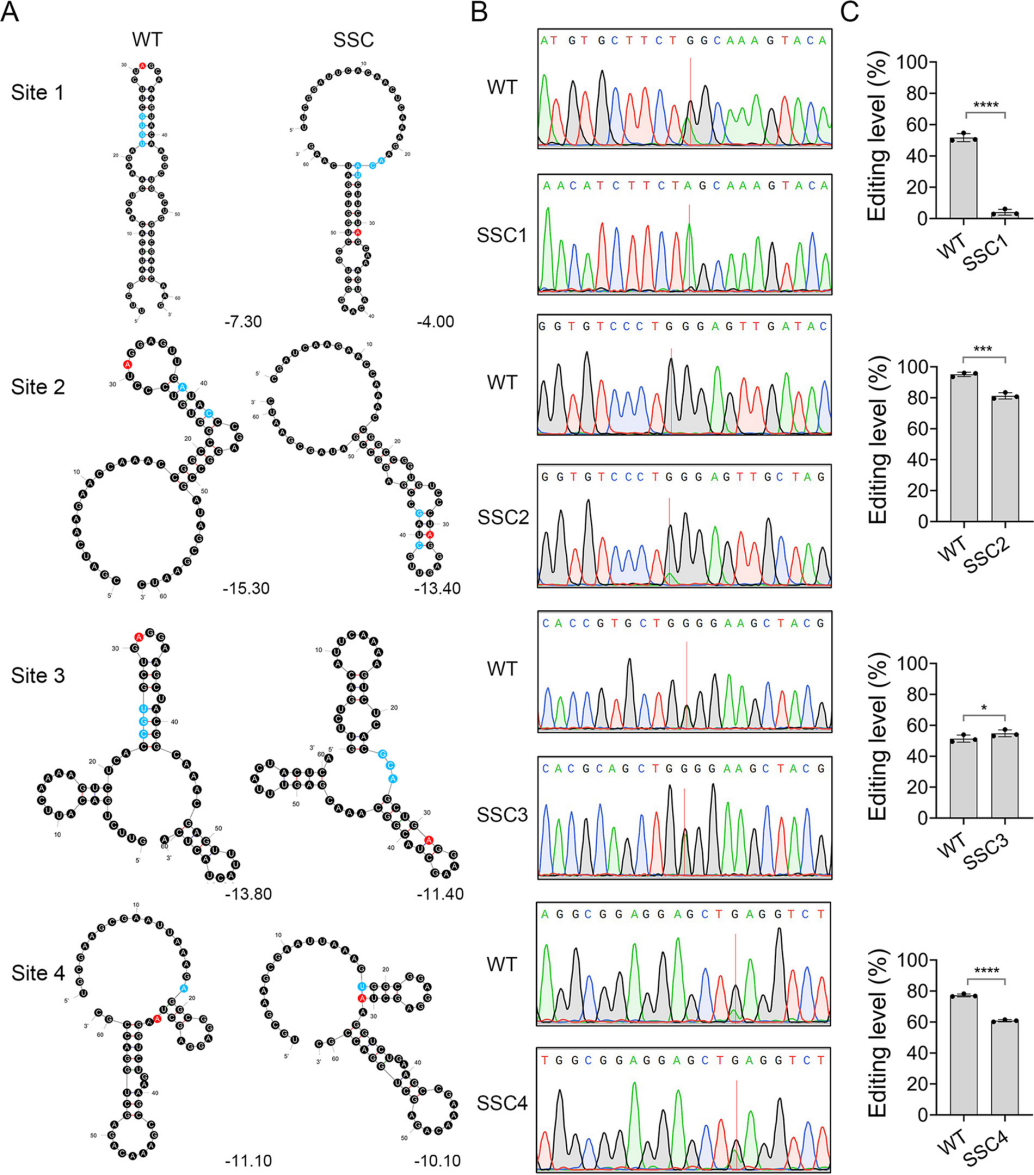
Effect of secondary structures on RNA editing of FG3G34330. (A) Predicted RNA secondary structures for wild type (WT) and secondary structure change (SSC) sequences of FG3G34330 containing the editing sites together with their upstream and downstream 30 nt sequences. Red, editing sites; Blue, mutation sites for secondary structure changes. The minimum free energy (MFE) value of each predicated RNA structure is indicated. (B) Sequencing traces of the 4 editing sites and their flanking sequences amplified from RNA isolated from perithecia of WT and marked mutants. Red lines mark the editing sites with mixed peaks of A and G although only the dominating peak is shown in the sequence on the top. (C) Means and standard deviations of the editing levels were estimated from three biological replicates (*n* = 3). Significant differences for pairwise comparison are based on *t* test (*, *P < *0.05; ***, *P < *0.001; ****, *P < *0.0001).

When the upstream −30 nt and downstream 30 nt sequences of 4 editing sites of FG3G34330 were used to predict RNA secondary structures, the editing sites 1 to 3 were situated in the hairpin loop while the editing site 4 was in the multi-loop (Fig. 4A). To investigate the influence of RNA secondary structures on editing efficiency, we introduced several mutations upstream or downstream of the editing sites that change the RNA loops bearing the editing sites into stems in their secondary structures (Fig. 4A). All the mutations were designed with minimum effects on the MFE of the secondary structures bearing editing sites. In comparison with the wild type, the editing levels of sites 1, 2, and 4 were significantly reduced in the secondary structure change (SSC) mutants (*t* test, *P < *0.05) (Table S3). Particularly, the editing level of site 1 was reduced over 12 folds (Fig. 4B and C). These results indicate that RNA secondary structures contribute importantly to the editing efficiency. Unexpectedly, mutations resulting in the hairpin loop to stem change slightly increased the editing level of site 3, suggesting complex regulation patterns.

### Efficient editing also depends on the full-length mRNA molecule of FG3G34330.

**FIG 5:**
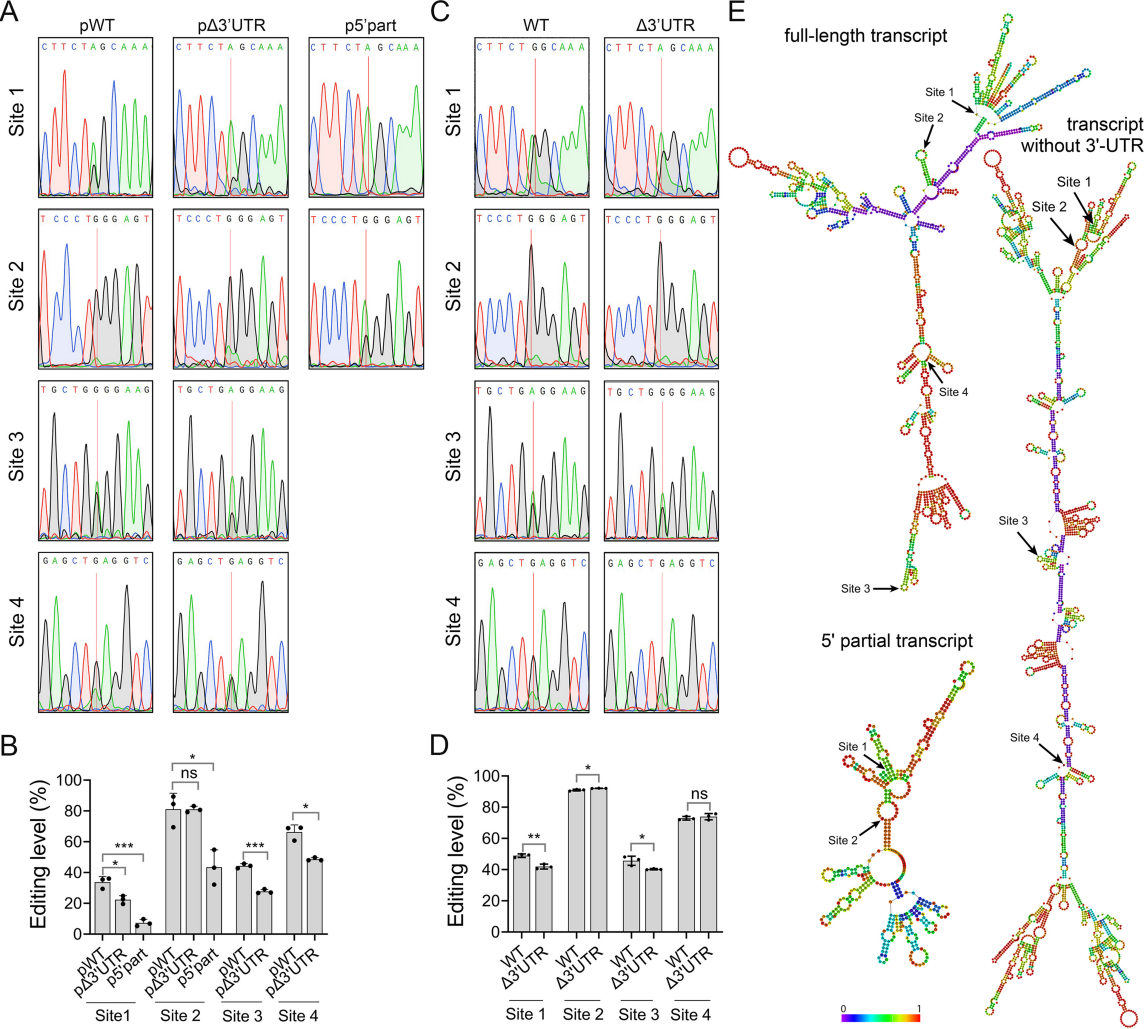
Effect of full-length mRNA molecule on RNA editing of FG3G34330. (A) and (C) Sequencing traces for flanking sequences of each editing site of FG3G34330 amplified from RNA isolated from perithecia of wild type (WT), transformants ectopically expressing the full-length transcript (pWT) or transcript without 3′-untranslated region (UTR) (*p*Δ3’UTR) or with only the 5′-UTR and the first 137 bp coding sequences (p5’part), and *in situ* 3′ UTR deletion mutant (Δ3’UTR). Red lines mark the editing sites with mixed peaks of A and G although only the dominating peak is shown in the sequence on the top. (B) and (D) Means and standard deviations of the editing levels were estimated from 3 biological replicates (*n* = 3). Significant differences for pairwise comparison are based on *t* test (*, *P < *0.05; **, *P < *0.01; ***, *P < *0.001; ns, not significant). (E) RNA secondary structure plots of the full-length transcript (−330 to 1278 bp relative to translational initiation), transcript without 3′-UTR (−333 to 1047 bp), and 5′ partial transcript (−330 to 137 bp) of FG3G34330. Colors represent the base-pair probabilities. The 4 A-to-I editing sites are indicated.

To determine whether efficient RNA editing depends on intact mRNA molecules, we generated transformants ectopically expressing the full-length and 5′-partial (including the 5′ untranslated region [5′-UTR] and the first 137 bp coding sequences) transcripts of FG3G34330 (Table S3). In comparison with the transformants expressing the full-length transcript, the editing levels of both sites 1 and 2 located in the 5′-partial transcript variant were dramatically reduced (Fig. 5A and B).

To further assay the influence of the intact RNA molecule on the editing, we generated transformants ectopically expressed the FG3G34330 transcript without 3′-UTR. The editing levels of all sites except site 2 were significantly reduced in the transformants ectopically expressing the FG3G34330 transcript without 3′-UTR (*t* test, *P < *0.05) (Fig. 5A and B). To rule out the possibility of the potential influence of ectopic expression, we deleted the 3′-UTR of FG3G34330 at the native locus and then measured the editing levels of the 4 editing sites in the FG3G34330^Δ3’UTR^ mutant. Removing the 3′-UTR *in situ* did not affect the editing level of site 4 but slightly reduced the editing levels of sites 1 and 3 (Fig. 5C and D). Site 2 had a slight increase in its editing level in the mutant. These results suggest that different lengths of RNA molecules affect editing efficiency.

To determine whether the influence of the different lengths of RNA molecules on editing is caused by alterations of secondary structure elements surrounding the editing sites, we predicted the secondary structures for the full-length transcript (−330 to 1278 bp relative to translational initiation), transcript without 3′-UTR (−333 to 1047 bp), and 5′ partial transcript (−330 to 137 bp) of FG3G34330. In the predicted secondary structures of the full-length transcript, editing sites 2 and 3 were situated in the hairpin loop while the editing sites 1 and 4 were in the multi-loop (Fig. 5E). In both the transcript without 3′-UTR and 5′ partial transcript of FG3G34330, the predicated multi-loop bearing editing site 1 was changed into the stem and the predicated hairpin loop bearing editing site 2 was changed into multi-loop. Changes in RNA secondary structure elements bearing the 2 editing sites did not readily explain the changes in their editing levels in both transcript variants. In addition, the predicted secondary structure elements bearing editing sites 3 and 4 were not changed although their editing levels were reduced in the transcript without 3′-UTR of FG3G34330. Therefore, editing level alterations observed in different transcript variants of FG3G34330 may not be the result of RNA secondary structure changes.

We then assayed the expression of different FG3G34330 alleles. The transcript without 3′-UTR and 5′ partial transcript had reduced expression levels relative to the full-length transcript in ectopically expressed transformants (Fig. S5). Since there was no obvious positive correlation between RNA editing level and gene expression level (9), it is not clear whether the decreased editing level of these truncated transcripts in the ectopically expressed transformants is caused by the decrease in transcript expression. However, the expression level of the transcript without 3′-UTR in the *in situ* mutant FG3G34330^Δ3’UTR^ had no significant change compared to the wild type (*t* test, *P > *0.05) (Fig. S5). Therefore, additional elements in the full-length transcript of FG3G34330 may regulate editing efficiency as well.

10.1128/mbio.01872-22.5 FIG S5 Relative expression of FG3G34330 alleles in PH-1 (WT), transformants ectopically expressing the full-length transcript (pWT) or transcript without 3’-untranslated region (UTR) (pΔ3’UTR) or with only the 5’-UTR and the first 137 bp coding sequences (p5’part), and *in situ* 3’ UTR deletion mutant (Δ3’UTR). The gene expression level in WT was arbitrarily set to 1. Means and standard deviations of relative expression levels were calculated from 3 biological replicates. Significant differences for pairwise comparison are based on *t*-test (**, *P < *0.01; ****, *P < *0.0001; ns, not significant). Download FIG S5, TIF file, 0.3 MB. Copyright © 2022 Feng et al. 2022 Feng et al. https://creativecommons.org/licenses/by/4.0/ This content is distributed under the terms of the Creative Commons Attribution 4.0 International license.

### Machine learning models accurately predict genome-wide editing sites but not editing levels from sequence and secondary structure features.

**FIG 6:**
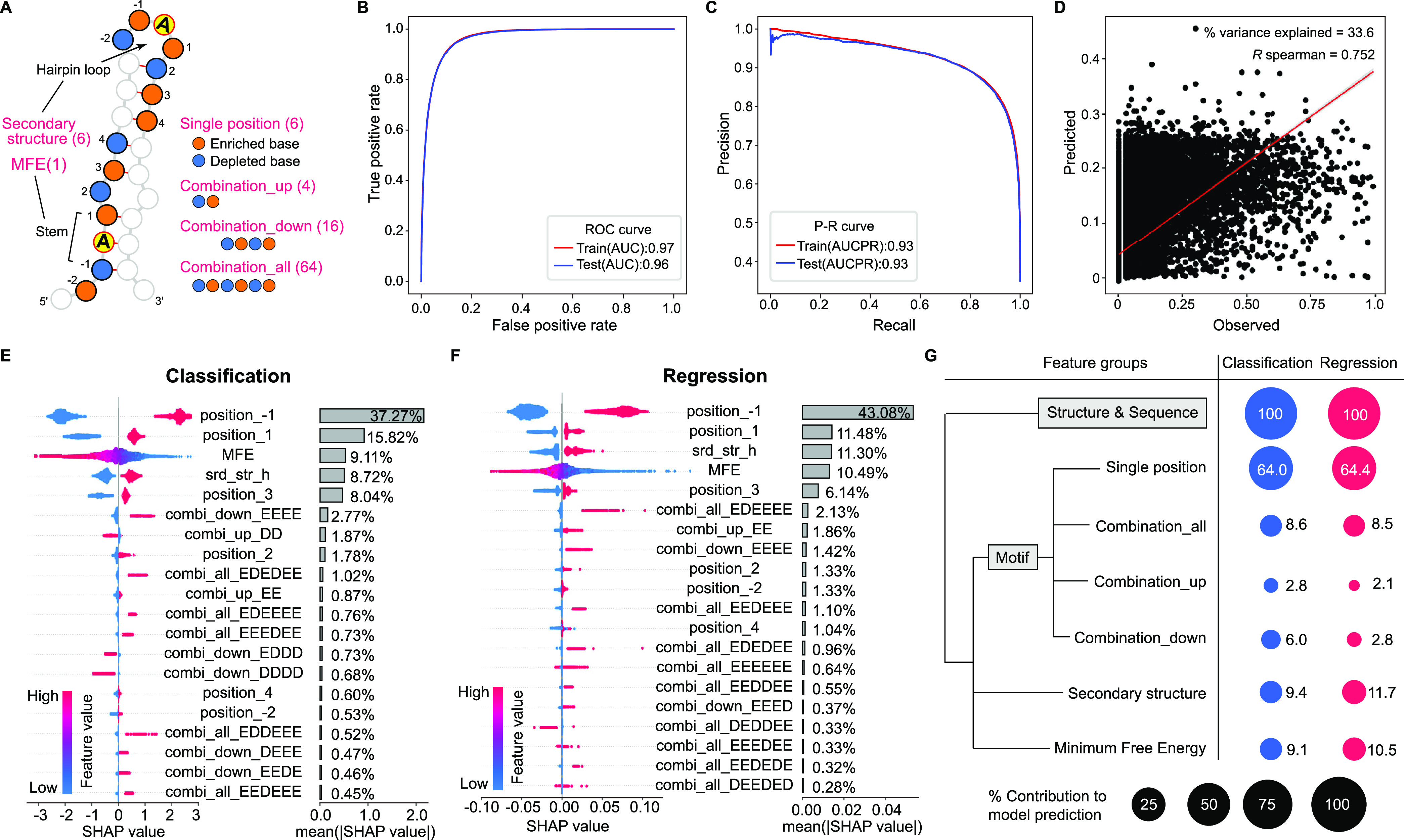
Machine learning model predictions and quantitative contributions of *cis*-regulatory features to the model prediction. (A) Graphical representation of the RNA sequence and structural features of editing or non-editing A sites used for XGBoost analysis. The features are categorized into 6 groups (shown in red font). Numbers in parentheses indicate the number of features in each group. The individual features included in each feature group are listed in Table S4. (B) and (C) Receiver Operating Characteristic (ROC) (B) and Precision-Recall (P-R) (C) curves of the classification model prediction of RNA editing site using XGBoost in training data set and held-out test data set. AUC, Area under the ROC Curve; AUCPR, Area under the Precision-recall Curve. (D) Editing level predictions in held-out test data set using XGBoost with a regression model. R^2^ is a measure of the % variance explained. Spearman R indicates a correlation between observed and predicted editing levels. Error bands (in gray) the 95% pointwise confidence bound for the mean predicted value, using linear smoothing. (E) and (F) SHAP values for the 20 most important features driving XGBoost predictions with classification (E) and regression models (F). Each dot indicates a site in the held-out test data set and the dot color shows the SHAP value from high (red) to low (blue). Positive and negative SHAP values indicate the features that drive the prediction over and below the data set base value, respectively. Features are ranked from top (most significant) to bottom (least significant) by predictive importance. The percent contribution of individual features to model prediction is indicated. The capital letters “E” and “D” in the feature names represent the enriched and depleted nucleotides at −2 to +4 positions of editing sites. (G) Contributions of different feature groups to the prediction of editing sites (by classification model) and levels (by regression model). Black dots indicate the scale.

To quantitatively capture the complex relationship between the multidimensional features of RNA sequences and secondary structures and the specificity and efficiency of editing, we turned to machine learning models. A set of 97 features categorized into 6 groups was used to annotate the RNA sequence and secondary structural elements surrounding each of the editing sites and the randomly selected non-edited A sites (negative sample) (Fig. 6A and Table S5). A classification model and a regression model were trained on these sites via the XGBoost algorithm (24) to map the feature annotations to corresponding binarized labels (edited versus not edited) and editing levels, respectively. To evaluate the prediction performance of our models, we trained and tuned models on a subset of sites and then tested model performance on a held-out test set of sites. Editing sites were predicted accurately by our classification model (area under the curve, AUC = 0.96; area under the precision-recall curve, AUCPR = 0.93) (Fig. 6B and C). However, the regression model yielded lower predictive performance for editing levels. Whereas the predicted editing levels were well correlated with the observed editing levels for the sites in the held-out test set with a Spearman correlation coefficient (R_s_) of 0.75, the model only accounted for 33.8% of the variance (R^2^) in editing levels (Fig. 6D). Therefore, it is possible to predict genome-wide RNA editing sites but not editing levels with high accuracy from the discovered sequence and secondary structure features using our machine learning models.

10.1128/mbio.01872-22.10 TABLE S5 Summary of feature information used for Machine learning. Download Table S5, DOCX file, 0.03 MB. Copyright © 2022 Feng et al. 2022 Feng et al. https://creativecommons.org/licenses/by/4.0/ This content is distributed under the terms of the Creative Commons Attribution 4.0 International license.

10.1128/mbio.01872-22.9 TABLE S4 Summary of primers used. Download Table S4, DOCX file, 0.02 MB. Copyright © 2022 Feng et al. 2022 Feng et al. https://creativecommons.org/licenses/by/4.0/ This content is distributed under the terms of the Creative Commons Attribution 4.0 International license.

### Model interpretation provides insights into the quantitative contributions of features to the RNA editing predictions.

To quantify the contribution of each feature to the model prediction in the held-out test sets, we used the TreeExplainer SHAP (Shapley Additive exPlanations) algorithm (25) to interpret our classification and regression models. The SHAP scores of features demonstrate the directionality of predictive association of the features with RNA editing sites or levels. For each model, we summarized the top 20 features based on their relative importance for driving model prediction and plotted their SHAP scores for the sites in the held-out test set (Fig. 6E and F). Undoubtedly, nucleotide preference (enriched versus depleted) at the −1 position was the strongest contributor for driving model predictions for both editing sites and levels, followed by the nucleotide preference at the +1 position. The relative importance of nucleotide preference at different positions was ranked as −1 (37.27%) > +1 (15.82%) > +3 (8.04%) > +2 (1.78%) > +4 (0.60%) > −2 (0.53%) for editing site prediction and −1 (43.08%) > +1 (11.48%) > +3 (6.14%) > +2 (1.33%) = −2 (1.33%) > +4 (1.04%) for editing level prediction. These results indicate that the relative importance of nucleotide preference at individual positions is generally consistent for the prediction of editing sites and levels.

The third to fourth ranking features were the hairpin loop structure (srd_str_h) and the MFE (Fig. 6E and F). The hairpin loop structure had a slightly higher relative contribution than MFE for editing level prediction (11.30% versus 10.49%), but the opposite for editing site prediction (8.72% versus 9.11%). The relative importance of different secondary structure elements was ranked as hairpin loop (8.72%) > interior loop (0.24%) > stem (0.19%) > multi-loop (0.12%) > free (0.11%) for editing site prediction and hairpin loop (11.30%) > stem (0.22%) > multi-loop (0.10%) > interior loop (0.08%) > free (0.03%) for editing level prediction. Therefore, for both model predictions, the contribution of the other 4 types of secondary structure elements is minor relative to the hairpin loop.

The combination of enriched nucleotides at downstream +1 to +4 positions (combi_down_EEEE) had a high contribution for both editing site prediction (2.77%, sixth ranking feature) and editing level prediction (1.42%, eighth ranking feature). However, a combination of depleted nucleotides at upstream −2 and −1 positions (combi_up_DD) had a high contribution for editing site prediction (1.87%, seventh ranking feature), but contributed less to editing level prediction (out of the top 20 features) (Fig. 6E and F). In addition, the relative importance of the features in the combination of all 6 positions group (Combination_all) is variable for the prediction of editing sites and levels. For example, the combination of depleted nucleotides at −1 and +2 positions and enriched nucleotides at −2, +1, +3, and +4 positions (combi_all_EDEDEE) was the most important feature for editing site prediction (1.02%, ninth ranking feature) while the combination of depleted nucleotides at −1 position and enriched nucleotides at the other 5 positions (combi_all_EDEEEE) was the most important feature for editing level prediction (2.13%, sixth ranking feature). These results suggest that the combination modes of neighboring nucleotides have distinct effects on the editing specificity and efficiency.

By integrating the contributions of individual features in each feature group, we found that the relative importance of different feature groups was consistent for the prediction of the editing sites and levels (Fig. 6G). The nucleotide preferences at individual positions (Single position) had the largest contribution (64.0% for both models), followed by secondary structure (9.4% for sites and 11.7% for levels) and MFE (9.1% for sites and 10.5% for levels). Combinations of different nucleotides at all 6 positions (Combination_all) were also important (8.6% for sites and 8.5% for levels). Notably, the combination of nucleotides at downstream 4 positions (Combination_down) is more important for editing site prediction (6.0%) but less important for editing level prediction (2.8%). Taken together, the systematic interpretation of our models demonstrates the promise of predictive *cis*-regulatory models of RNA editing and highlights the need for comprehensive knowledge of *cis*-regulatory features to learn more accurate models of RNA editing.

## DISCUSSION

We have previously identified 26,056 A-to-I editing sites in F. graminearum (8). In this report, we expanded the number of identified A-to-I editing sites to 40,235 and combined genome-wide comparisons, proof-of-concept experiments, and machine learning to decipher *cis*-regulatory elements that affect A-to-I RNA editing in F. graminearum. We identified a variety of *cis*-sequence features important for the specificity and efficiency of RNA editing. Nucleotide preferences (preferred and depleted nucleotides) at −2 to +4 positions of editing sites are the most important features for editing specificity and efficiency, especially the nucleotide preferences at the −1 position. In animals, although adjacent nucleotides beyond nearest neighbors also affect editing specificity, the 5′ and 3′ nearest neighbors are most influential (26). Similar to fungi, the 5′ nearest neighbor has more influence on editing than the 3′ nearest neighbor in animals (26). However, unlike in fungi, the 5′ and 3′ nearest neighbors play a role only in editing specificity rather than efficiency and the effect of the 5′ nearest neighbor is independent of the 3′ nearest neighbor in animals (26, 27). In F. graminearum, not only the number of positions with preferred nucleotides but also the combination of preferred nucleotides with depleted ones is important for editing. In addition, some *cis*-sequence features have different importance for editing specificity and efficiency. For example, the combination of upstream depleted nucleotides (−2 and −1 positions) has a more important role in editing specificity than in efficiency.

Besides primary sequence features, we also identified the RNA secondary structure features important for A-to-I RNA editing in F. graminearum. Unlike in animals where A-to-I editing occurs in dsRNA (stem) structure, the A-to-I editing in F. graminearum preferentially targets the A’s located in hairpin loops. The editing levels of the sites located in hairpin loops are significantly higher than those in other types of RNA secondary structures. The more stable (lower MFE value) the hairpin loop structures, the higher the editing levels. Despite their importance, the roles of RNA secondary structures in editing are less important compared with primary sequences. This is the opposite of what happens in animals, where the editing site structure and its stability have the largest contributions to editing efficiency (15). The different aspects of RNA editing recognition may reflect the distinct editing machinery between fungi and animals. In addition, we found that the editing sites with less preferred neighboring sequences were more likely located in hairpin loops, suggesting that the hairpin loop structure is more important for editing the A’s with less preferred neighboring sequences. The primary sequence and secondary structure may be cooperative for RNA editing.

Our machine learning models built from the discovered RNA sequence and secondary structural features can accurately predict genome-wide editing sites but not editing levels. The lower performance of our current model for genome-wide editing level prediction is not surprising considering the diversity of the substrates. In fact, in animals, machine learning models can accurately predict substrate-specific RNA editing levels but do not generalize across substrates (15). These results indicate that the *cis*-regulatory principle of editing efficiency is more complex than that of editing specificity. Consistent with this, the intact RNA molecule of FG3G34330 was found to be important for editing efficiency. Because true RNA molecule is rarely two-dimensional *in vivo*, tertiary structural elements likely determine the RNA editing efficiency. In animals, RNA tertiary structure has been proposed to regulate A-to-I editing (28–30). It remains to be seen what and how RNA tertiary structures regulate A-to-I editing in fungi. Alternatively, the low performance for editing level prediction could be caused by limitations in RNA structure prediction. Currently, *in silico* prediction of correct RNA structures is still confronted with a major challenge. The *in vivo* RNA structures can be affected by RNA binding proteins and ATP-dependent helicases (31). The thermodynamic forces that drive RNA folding *in vitro* may not be sufficient to predict *in vivo* RNA structures.

In summary, our work uncovers multiple *cis*-regulatory elements and their effects on A-to-I RNA editing in F. graminearum, highlighting the complexity of the *cis*-regulatory principles of editing efficiency in fungi. Understanding the *cis*-regulatory principles of A-to-I RNA editing will help us to elucidate the editing mechanism and develop a model that accurately predicts editing sites and levels in new fungal genomes.

## MATERIALS AND METHODS

### Strains and cultural conditions.

The wild type strain PH-1 of F. graminearum (32) and its mutants generated in this study were routinely cultured on potato dextrose agar (PDA) plates at 25°C. For sexual reproduction, 5-day-old aerial hyphae on 400 g/L carrot agar plates were pressed down with 800 μL of 0.1% Tween 20 and incubated under black light. Protoplast preparation and transformation were performed as described previously (33). For transformant selection, Top medium (0.3% yeast extract, 0.3% Casamino Acids, and 20% sucrose) was added with 300 μg/mL hygromycin B (H005, MDbio, China), 25 μg/mL 5-fluoro-2’-deoxyuridine (Floxuridine) (HY-B0097, MCE), or 150 μg/mL Geneticin (Sigma-Aldrich).

### Strand-specific RNA-seq.

Perithecia of PH-1 were collected from mating cultures at 6-dpf with 5 biological replicates. Total RNA of each sample was extracted with the RNAprep Pure Plant Kit (Tiangen Biotech), and poly (A)+ mRNA was enriched with oligo (dT) magnetic beads. Strand-specific RNA-seq libraries were prepared with the NEBNext Ultra Directional RNA Library Prep Kit following the manufacturer’s instruction and sequenced by Illumina HiSeq-2500 with a 2 × 150-bp paired-end read mode at the Novogene Bioinformatics Institute. For each library, more than 20 Mb of high-quality reads were obtained. The RNA-seq reads were submitted to the NCBI SRA database under accession numbers included in Table S1.

### Identification and analysis of A-to-I RNA editing sites.

Published RNA-seq and DNA-seq data of PH-1 generated in our previous study (8) were downloaded from the NCBI SRA database under accession numbers included in Table S1. The most recent genome sequences of PH-1 (version YL1) (34) were obtained from FgBase (http://fgbase.wheatscab.com/). The DNA-seq and RNA-seq reads were mapped to the PH-1 genome using Bowtie 2 (35) and HISAT2 (36), respectively. Duplicated reads in the mapped BAM file were removed using the *MarkDuplicates* from Picard package v1.99 (http://broadinstitute.github.io/picard/). The unduplicated RNA-seq BAM files of all samples were combined into one BAM file and then divided into 2 separated files containing sense-strand and antisense-strand read alignments, respectively, using BamTools v2.4.0 (37). A-to-I RNA editing sites were identified by REDItools2 (38) using matched DNA-seq and RNA-seq data with an editing level cutoff value of 3% as described previously (9, 39). The numbers of different RNA variant sites detected were listed in Table S2. The identified A-to-I RNA editing sites are available at FgBase (http://fgbase.wheatscab.com/).

The nucleotide preference surrounding the A-to-I editing sites was visualized using WebLogo 3 (40) and Two Sample Logo (41). The secondary structures of both full-length and local RNA sequences were predicted using RNAFold (42). The random sampling of editable sites (control) with similar nucleotide preferences as edited sites was performed with the scripts developed in our previous study (https://github.com/wangqinhu/NC.edits).

### Targeted gene deletion, site-directed mutagenesis, and allelic exchanges.

The gene deletion construct expresses a translational fusion of the hygromycin phosphotransferase (HygR) with the thymidine kinase (TK) from herpes simplex virus 1, which confers resistance to the antibiotic hygromycin and sensitivity to nucleoside analog 5-fluoro-2’-deoxyuridine (Floxuridine) (43, 44). The split-marker approach (45) was used to generate the gene replacement construct for the FG3G34330 gene. About 1-kb upstream and 1-kb downstream fragments of the gene were amplified and connected to the N- and C-terminal regions of the selectable marker cassette by overlapping PCR, respectively. After the transformation of PH-1 protoplasts, hygromycin-resistant transformants were identified and confirmed by PCR assays.

Allelic fragments of the FG3G34330 gene with desired nucleotide mutations or sequence deletions were generated by overlapping PCR. For allelic exchange at the native locus, the allelic fragments containing about 1-kb upstream and 1-kb downstream homologous arms were transformed into the protoplasts of the FG3G34330 deletion mutant. Floxuridine-sensitive transformants were identified and desired mutations at the native locus were verified by sequencing analysis. Quantitative PCR was used to confirm the obtained mutants without ectopic integration. For ectopic expression of the mutant alleles of FG3G34330, the allelic genes under the control of the native promoter were cloned into the pFL2 plasmid by the yeast gap repair method (46). The constructs were confirmed by sequencing analysis and transformed into the *ΔFG3G34330* deletion mutant. Transformants resistant to Geneticin were identified and confirmed by PCR assays. All the mutant strains generated and primers used in this study were listed in Table S3 and Table S4, respectively.

### RT-PCR.

Total RNA was isolated with the TRIzol reagent (Invitrogen) from 6-dpf perithecia as described. For each strain, at least 3 biological replicates were prepared. cDNA synthesis was performed with FastQuant RT Kit (TIANGEN) following the manufacturer’s instructions. RT-PCR products were gel purified and subjected to direct sequencing. Sequencing of RT-PCR products was done using an automatic DNA sequencer. Relative expression levels of FG3G34330 alleles in different strains were assayed by quantitative real-time RT-PCR using the 2^-ΔΔCT^ method with the actin gene as an internal control. All the primers used were listed in Table S4.

### Quantitative analysis of editing level from Sanger sequencing traces.

The Sanger sequencing traces were visualized using SnapGene Viewer 4.3 (https://www.snapgene.com/snapgene-viewer/). Quantitative analysis of editing level was performed using QSVanalyser (47), which calculates relative proportions of A/G variants at the editing site from the Sanger sequence trace based on normalized peak heights. Means and standard deviations of editing levels were calculated from 3 biological replicates.

### Machine learning models of RNA editing.

Both the classification model and the regression model were constructed by XGBoost v1.6.0 (24). The non-editing A sites in the edited transcript were randomly selected as a negative sample. For XGBoost analysis, the ratio of the positive sample (all editing sites) to the negative sample is 1:2 in the data set. The sequence and structural features for each site were extracted and included in a feature matrix. The matrix was randomly separated into 2 splits: training on 80% of sites and testing on the remaining 20%. The classification model was trained until no reduction in error rates on the test split, and the regression model was trained until no reduction in root mean square error (RMSE) on the test split. The SHAP v0.40.0 (25) was applied to interpret feature importance from the XGBoost model. SHAP scores were computed for each feature as a measure of feature importance using the “shap_values” function within the“TreeExplainer” class. Features were ranked by their mean absolute value of SHAP scores across the test data set. The relative contribution (%) of each feature to the total was calculated. All feature extraction and model training code are available to access on GitHub: https://github.com/XINYUCAO111/machineleaning_of_RNA_editing.
