# Supplementary material for: Uncovering Cis-Regulatory Elements Important for A-to-I RNA Editing in Fusarium graminearum
Source: mBio. 2022 Sep 14;13(5):e01872-22. doi: 10.1128/mbio.01872-22 (PMC9600606; doi:10.1128/mbio.01872-22)
Supplement: TABLE S1 [file mbio.01872-22-s0006.docx]

**Table S1 RNA-seq and DNA-seq data used in this study.**

| Type of data | Library method | Sample name | Sample description | Reads length | Reads count | Mapping rates | Mapped reads | SRA_Run | Reference |
| --- | --- | --- | --- | --- | --- | --- | --- | --- | --- |
| Strand-specific RNA-Seq | poly(A) selection | Sex6d-1 | Perithecia from carrot agar plates 6 days post-fertilization | 2×150 bp | 42,369,260 | 97.63% | 41,365,108 | SRR17634834 | This study |
| Strand-specific RNA-Seq | poly(A) selection | Sex6d-2 | Perithecia from carrot agar plates 6 days post-fertilization | 2×150 bp | 43,146,242 | 98.52% | 42,507,677 | SRR17634833 | This study |
| Strand-specific RNA-Seq | poly(A) selection | Sex6d-3 | Perithecia from carrot agar plates 6 days post-fertilization | 2×150 bp | 26,460,398 | 96.57% | 25,552,806 | SRR17634832 | This study |
| Strand-specific RNA-Seq | poly(A) selection | Sex6d-4 | Perithecia from carrot agar plates 6 days post-fertilization | 2×150 bp | 29,352,656 | 98.23% | 28,833,113 | SRR17634831 | This study |
| Strand-specific RNA-Seq | poly(A) selection | Sex6d-5 | Perithecia from carrot agar plates 6 days post-fertilization | 2×150 bp | 23,989,382 | 96.72% | 23,202,530 | SRR12676630 | This study |
| Strand-specific RNA-Seq | rRNA depletion | Sex8d-1 | Perithecia from carrot agar plates 8 days post-fertilization | 2×150 bp | 37,139,802 | 97.22% | 36,107,316 | SRR2182499 | Liu et al, 2016, Genome Res. |
| Strand-specific RNA-Seq | rRNA depletion | Sex8d-2 | Perithecia from carrot agar plates 8 days post-fertilization | 2×150 bp | 41,287,404 | 96.77% | 39,953,821 | SRR2182501 | Liu et al, 2016, Genome Res. |
| DNA-Seq | / | PH-1_YL | Perithecia from carrot agar plates 7 days post-fertilization | 2×101 bp | 33,466,822 | 99.32% | 33,239,248 | SRR3030980 | Liu et al, 2016, Genome Res. |

Liu H, Wang Q, He Y, Chen L, Hao C, Jiang C, Li Y, Dai Y, Kang Z, Xu JR. 2016. Genome-wide A-to-I RNA editing in fungi independent of ADAR enzymes. *Genome Res* 26(4): 499-509.
