# Supplementary material for: Uncovering Cis-Regulatory Elements Important for A-to-I RNA Editing in Fusarium graminearum
Source: mBio. 2022 Sep 14;13(5):e01872-22. doi: 10.1128/mbio.01872-22 (PMC9600606; doi:10.1128/mbio.01872-22)
Supplement: TABLE S2 [file mbio.01872-22-s0007.docx]

**Table S2 Number of different RNA variant sites detected in *Fusarium graminearum*.**

| Type of variants | Number of sites |
| --- | --- |
| A→C | 44 |
| A→G | **40,235** |
| A→T | 35 |
| C→A | 24 |
| C→G | 13 |
| C→T | 43 |
| G→A | 122 |
| G→C | 39 |
| G→T | 64 |
| T→A | 54 |
| T→C | 324 |
| T→G | 37 |
| Total | 41,034 |
