# Supplementary figures and images for: Uncovering Cis-Regulatory Elements Important for A-to-I RNA Editing in Fusarium graminearum
Source: mBio. 2022 Sep 14;13(5):e01872-22. doi: 10.1128/mbio.01872-22 (PMC9600606; doi:10.1128/mbio.01872-22)

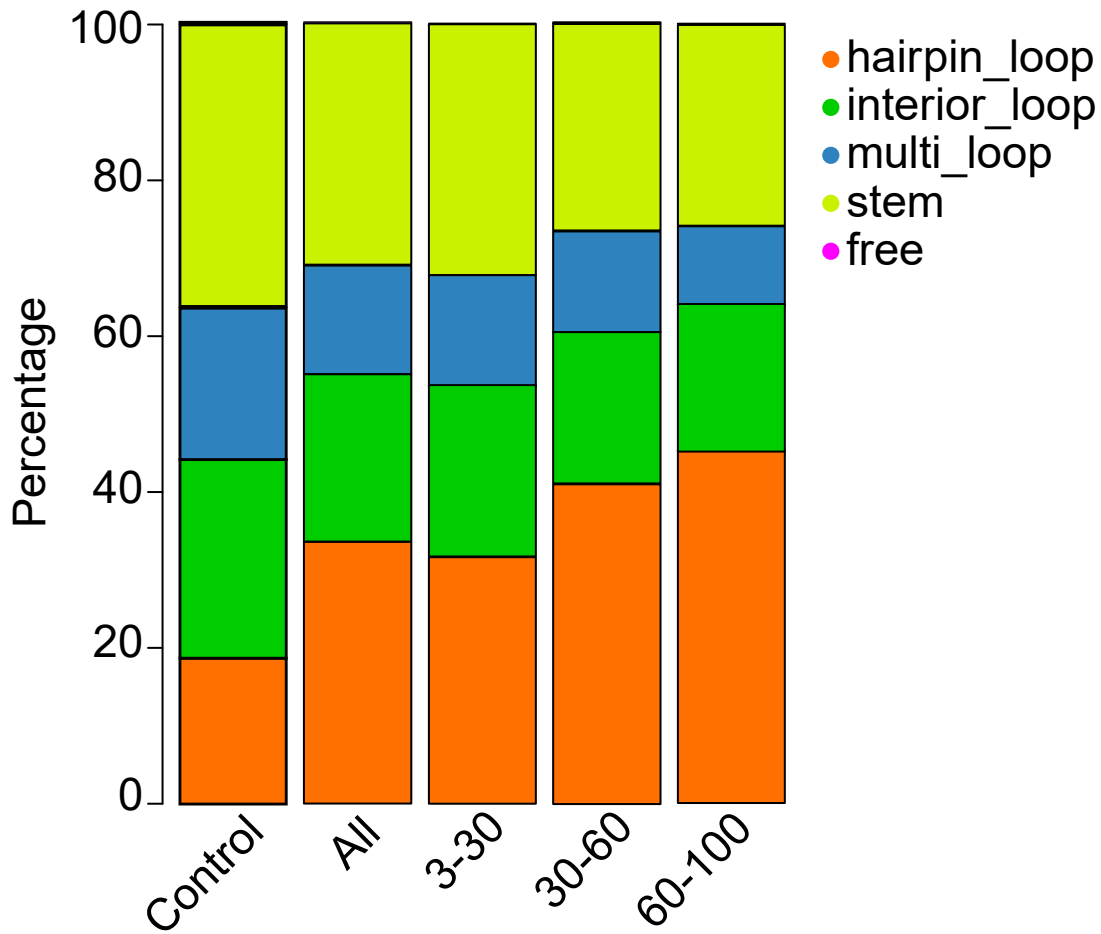

Supplement: FIG S1 [file mbio.01872-22-s0001.pdf]

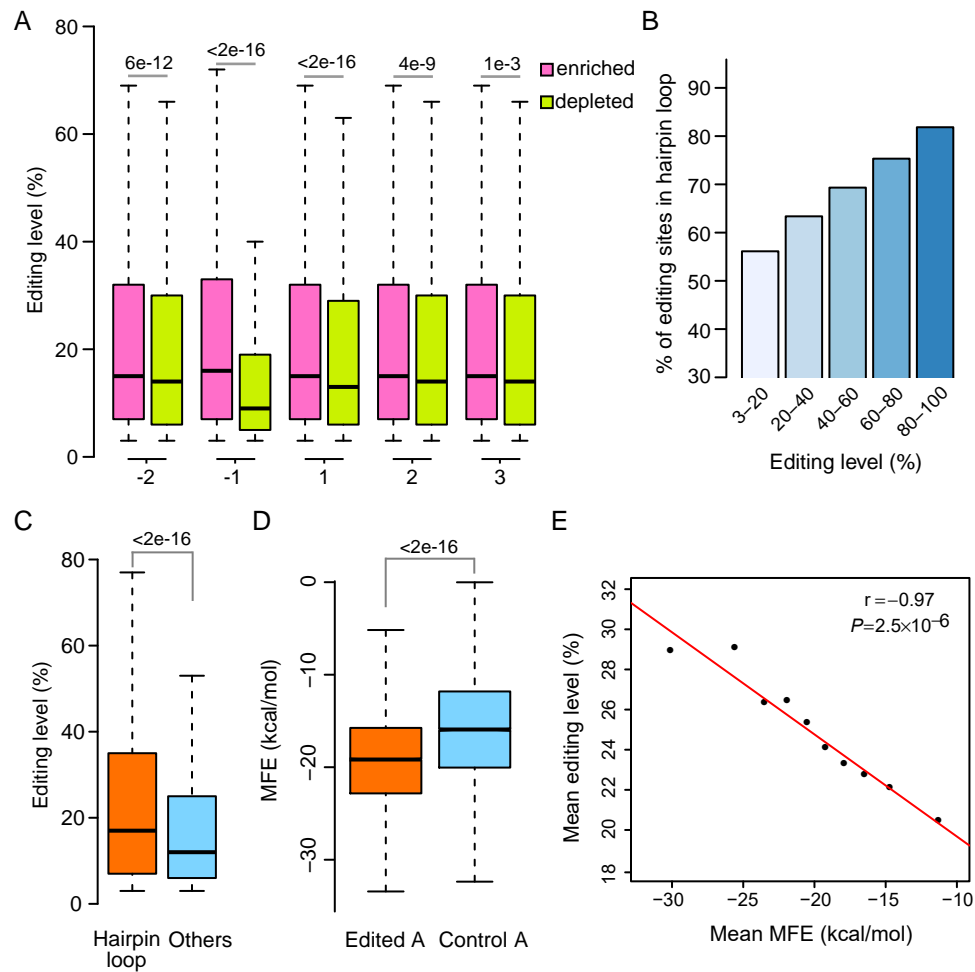

Supplement: FIG S2 [file mbio.01872-22-s0002.pdf]

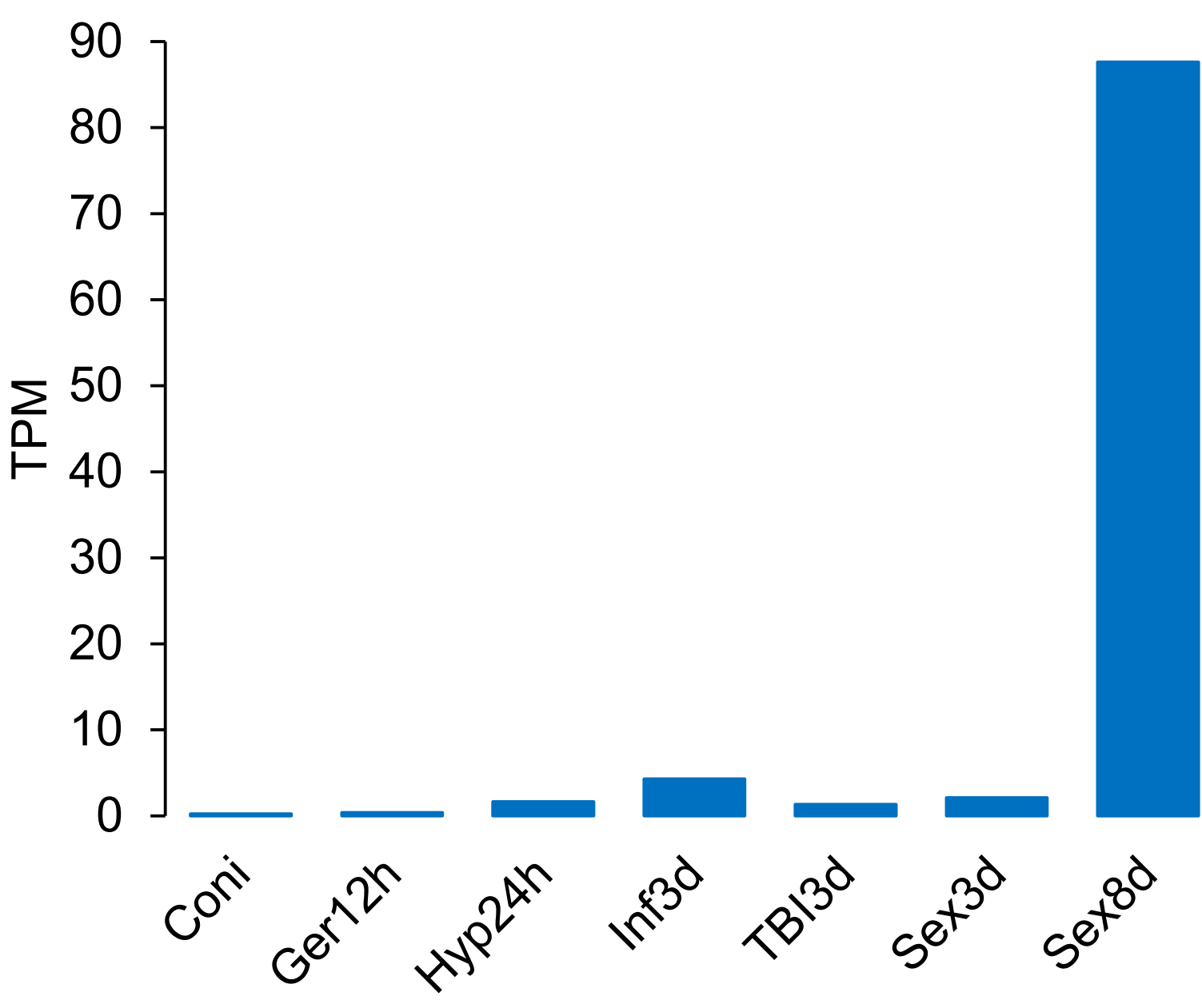

Supplement: FIG S3 [file mbio.01872-22-s0003.pdf]

WT

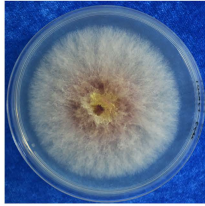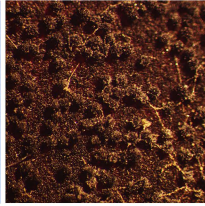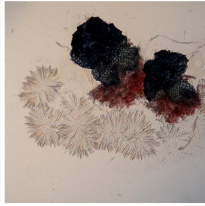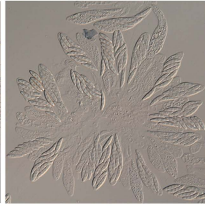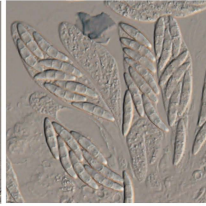

$\Delta$ FG3G34330

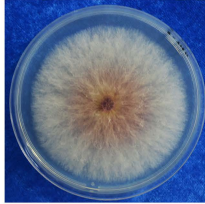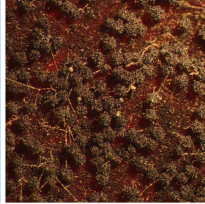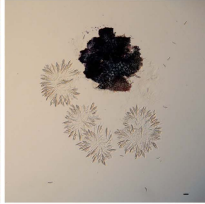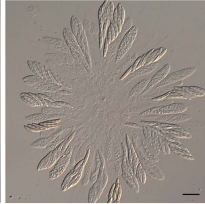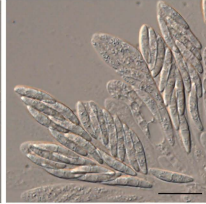

Supplement: FIG S4 [file mbio.01872-22-s0004.pdf]

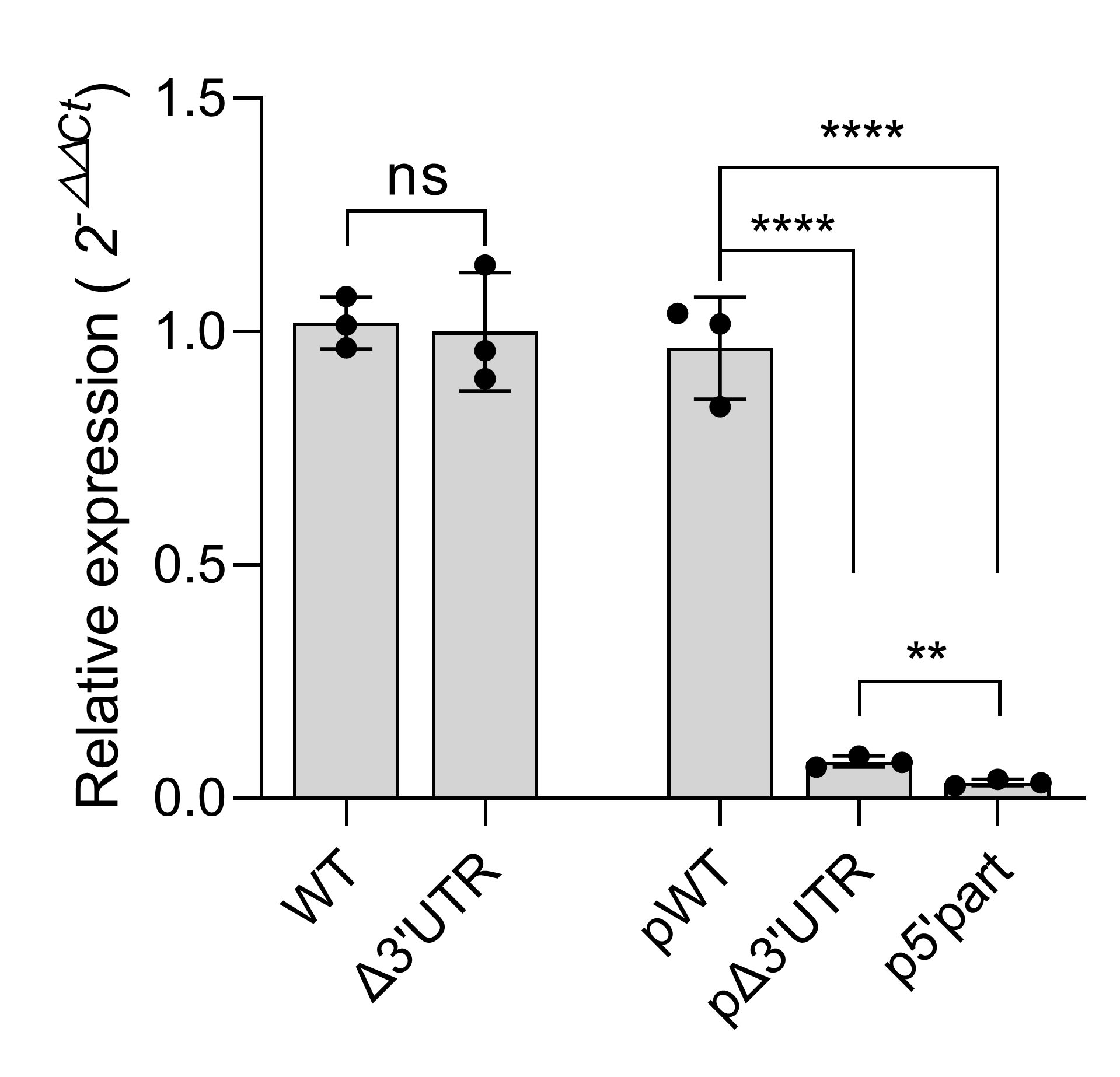

Supplement: FIG S5 [file mbio.01872-22-s0005.tif]
