# Supplementary material for: Uncovering Cis-Regulatory Elements Important for A-to-I RNA Editing in Fusarium graminearum
Source: mBio. 2022 Sep 14;13(5):e01872-22. doi: 10.1128/mbio.01872-22 (PMC9600606; doi:10.1128/mbio.01872-22)
Supplement: TABLE S3 [file mbio.01872-22-s0008.docx]

**Table S3** **Summary of strains used in this study.**

| Strain | Brief description | Reference |
| --- | --- | --- |
| PH-1 | Wild-type strain | Cuomo *et al*. (2007) |
| ΔFG3G34330-1, 8 | FG3G34330 deletion mutants | This study |
| 330-1(-1)-1 | FG3G34330-editing site 1^T(-1)G^ mutant | This study |
| 330-1(+2)-1 | FG3G34330-editing site 1^C(+2)G^ mutant | This study |
| 330-2(-2)-1 | FG3G34330-editing site 2^C(-2)G^ mutant | This study |
| 330-2(-1)-1 | FG3G34330-editing site 2^T(-1)G^ mutant | This study |
| 330-2(+1)-1 | FG3G34330-editing site 2^G(+1)C^ mutant | This study |
| 330-2(+2)-1, 3 | FG3G34330-editing site 2^G(+2)A^ mutants | This study |
| 330-2(+3)-1, 2, 3 | FG3G34330-editing site 2^A(+3)C^ mutants | This study |
| 330-3(-1)-1 | FG3G34330-editing site 3^G(-1)T^ mutant | This study |
| 330-4(-2)-7, 8, 9 | FG3G34330-editing site 4^C(-2)G^ mutants | This study |
| 330-4(-1)-1 | FG3G34330-editing site 4^T(-1)G^ mutant | This study |
| 330-4(+1)-4, 6 | FG3G34330-editing site 4^A(+1)C^ mutants | This study |
| 330-4(+2)-1 | FG3G34330-editing site 4^G(+2)A^ mutant | This study |
| 330-4(+3)-15, 16 | FG3G34330-editing site 4^G(+3)C^ mutants | This study |
| 330-2(-2,+1)-1 | FG3G34330-editing site 2^C(-2)G,G(+1)C^ mutant | This study |
| 330-2(-2,+2)-1 | FG3G34330-editing site 2^C(-2)G,G(+2)A^ mutant | This study |
| 330-2(-2,+3)-1, 2 | FG3G34330-editing site 2^C(-2)G,A(+3)C^ mutants | This study |
| 330-2(+1,+2)-1 | FG3G34330-editing site 2^G(+1)C,G(+2)A^ mutant | This study |
| 330-2(+1,+3)-1, 2 | FG3G34330-editing site 2^G(+1)C,A(+3)C^ mutants | This study |
| 330-2(+2,+3)-1 | FG3G34330-editing site 2^G(+2)A,A(+3)C^ mutant | This study |
| 330-4(-2,+2)-2, 3 | FG3G34330-editing site 4^C(-2)G,G(+2)A^ mutants | This study |
| 330-4(-2,+3)-1 | FG3G34330-editing site 4^C(-2)G,G(+3)C^ mutant | This study |
| 330-4(+2,+3)-1 | FG3G34330-editing site 4^G(+2)A,G(+3)C^ mutant | This study |
| 330-SSC1-1, 3 | FG3G34330 mutants with secondary structural change of editing site 1 | This study |
| 330-SSC2-3 | FG3G34330 mutant with secondary structural change of editing site 2 | This study |
| 330-SSC3-2 | FG3G34330 mutant with secondary structural change of editing site 3 | This study |
| 330-SSC4-6, 8, 9 | FG3G34330 mutants with secondary structural change of editing site 4 | This study |
| 330-Δ3'UTR-1, 2, 3 | FG3G34330-3'UTR (-) mutants | This study |
| 330-pWT-2, 2N | ΔFG3G34330/FG3G34330-3'UTR (+) transformants | This study |
| 330-pΔ3'UTR-2, 4 | ΔFG3G34330/FG3G34330-3'UTR (-) transformants | This study |
| 330-p5'part-2, 10 | ΔFG3G34330/FG3G34330-400bp sequence (at the 5' end including editing site 1 and site2) transformants | This study |

Cuomo CA, Guldener U, Xu JR, Trail F, Turgeon BG, Di Pietro A, Walton JD, Ma LJ, Baker SE, Rep M, et al. 2007. The Fusarium graminearum genome reveals a link between localized polymorphism and pathogen specialization. *Science* 317(5843): 1400-1402.
