# Supplementary material for: Uncovering Cis-Regulatory Elements Important for A-to-I RNA Editing in Fusarium graminearum
Source: mBio. 2022 Sep 14;13(5):e01872-22. doi: 10.1128/mbio.01872-22 (PMC9600606; doi:10.1128/mbio.01872-22)
Supplement: TABLE S5 [file mbio.01872-22-s0010.docx]

**Table S5 Summary of feature information used for Machine learning.**

| Group | Feature | Classification model | Regression model | Classification model | Regression model |
| --- | --- | --- | --- | --- | --- |
|  |  | mean\|SHAP\| | mean\|SHAP\| | % contribution | % contribution |
| Single position  (Preferred or depleted nucleotides at each of the -2 to +4 positions of editing sites) | position_-2 | 0.030858789 | 0.00164249 | 0.53 | 1.33 |
|  | position_-1 | 2.161257 | 0.053288113 | 37.27 | 43.08 |
|  | position_1 | 0.91760844 | 0.014201127 | 15.82 | 11.48 |
|  | position_3 | 0.4665614 | 0.007590522 | 8.04 | 6.14 |
|  | position_4 | 0.034910094 | 0.001288583 | 0.60 | 1.04 |
|  | position_2 | 0.10314233 | 0.001648056 | 1.78 | 1.33 |
| Secondary structure  (Types of RNA secondary structure elements bearing the editing sites: h, hairpin loop; i, interior loop; m, multi loop; s, stem; f and t, free) | srd_str_f | 0.002280243 | 1.03E-05 | 0.04 | 0.01 |
|  | srd_str_h | 0.5056051 | 0.013978445 | 8.72 | 11.30 |
|  | srd_str_i | 0.013656713 | 9.76E-05 | 0.24 | 0.08 |
|  | srd_str_m | 0.006894821 | 0.000117744 | 0.12 | 0.10 |
|  | srd_str_s | 0.011178203 | 0.000269794 | 0.19 | 0.22 |
|  | srd_str_t | 0.004002547 | 2.40E-05 | 0.07 | 0.02 |
| Minimum Free Energy | MFE | 0.52827454 | 0.012982179 | 9.11 | 10.49 |
| Combination_up  (Combination of preferred (E) and depleted (D) nucleotides at -2 and -1 positions) | up2sites_DD | 0.108182825 | 0.000227651 | 1.87 | 0.18 |
|  | up2sites_DE | 0.000392683 | 2.02E-05 | 0.01 | 0.02 |
|  | up2sites_ED | 0.002421845 | 0 | 0.04 | 0.00 |
|  | up2sites_EE | 0.050326772 | 0.002295431 | 0.87 | 1.86 |
| Combination_down  (Combination of preferred (E) and depleted (D) nucleotides at +1 to +4 positions) | down4sites_DDDD | 3.97E-02 | 0.00017522 | 0.68 | 0.14 |
|  | down4sites_DDDE | 0.005082652 | 1.04E-05 | 0.09 | 0.01 |
|  | down4sites_DDED | 0.009846869 | 1.81E-05 | 0.17 | 0.01 |
|  | down4sites_DDEE | 0.006662072 | 3.57E-05 | 0.11 | 0.03 |
|  | down4sites_DEDD | 0.003102831 | 3.53E-05 | 0.05 | 0.03 |
|  | down4sites_DEDE | 0 | 1.24E-06 | 0.00 | 0.00 |
|  | down4sites_DEED | 0.001800544 | 7.76E-06 | 0.03 | 0.01 |
|  | down4sites_DEEE | 0.027498288 | 0.000209681 | 0.47 | 0.17 |
|  | down4sites_EDDD | 0.04204908 | 0.000222529 | 0.73 | 0.18 |
|  | down4sites_EDDE | 0.000822494 | 5.57E-05 | 0.01 | 0.05 |
|  | down4sites_EDED | 0.007232559 | 0.000260339 | 0.12 | 0.21 |
|  | down4sites_EDEE | 0.002129254 | 4.00E-05 | 0.04 | 0.03 |
|  | down4sites_EEDD | 0.000609029 | 4.48E-05 | 0.01 | 0.04 |
|  | down4sites_EEDE | 0.026849944 | 0.00016772 | 0.46 | 0.14 |
|  | down4sites_EEED | 0.016300103 | 0.000455697 | 0.28 | 0.37 |
|  | down4sites_EEEE | 0.16092916 | 0.001752849 | 2.77 | 1.42 |
| Combination_all  (Combination of preferred (E) and depleted (D) nucleotides at -2 to +4 positions) | all6sites_DDDDDD | 0.000650135 | 0 | 0.01 | 0.00 |
|  | all6sites_DDDDDE | 0 | 0 | 0.00 | 0.00 |
|  | all6sites_DDDDED | 0.003995196 | 2.91E-07 | 0.07 | 0.00 |
|  | all6sites_DDDDEE | 0.00300787 | 2.48E-06 | 0.05 | 0.00 |
|  | all6sites_DDDEDD | 0 | 0 | 0.00 | 0.00 |
|  | all6sites_DDDEDE | 0 | 0 | 0.00 | 0.00 |
|  | all6sites_DDDEED | 0.003101123 | 0 | 0.05 | 0.00 |
|  | all6sites_DDDEEE | 0 | 0 | 0.00 | 0.00 |
|  | all6sites_DDEDDD | 0.003211426 | 0 | 0.06 | 0.00 |
|  | all6sites_DDEDDE | 0.016011039 | 7.43E-06 | 0.28 | 0.01 |
|  | all6sites_DDEDED | 0.00015004 | 2.57E-06 | 0.00 | 0.00 |
|  | all6sites_DDEDEE | 0.010678234 | 3.20E-06 | 0.18 | 0.00 |
|  | all6sites_DDEEDD | 0 | 0 | 0.00 | 0.00 |
|  | all6sites_DDEEDE | 0 | 0 | 0.00 | 0.00 |
|  | all6sites_DDEEED | 0.000778854 | 4.96E-06 | 0.01 | 0.00 |
|  | all6sites_DDEEEE | 0.000593967 | 4.58E-06 | 0.01 | 0.00 |
|  | all6sites_DEDDDD | 0.00077871 | 4.52E-06 | 0.01 | 0.00 |
|  | all6sites_DEDDDE | 0.013770876 | 0.000120077 | 0.24 | 0.10 |
|  | all6sites_DEDDED | 0.00055421 | 5.81E-05 | 0.01 | 0.05 |
|  | all6sites_DEDDEE | 0.015369304 | 0.000405102 | 0.27 | 0.33 |
|  | all6sites_DEDEDD | 3.22E-04 | 1.07E-05 | 0.01 | 0.01 |
|  | all6sites_DEDEDE | 0.000824134 | 3.64E-05 | 0.01 | 0.03 |
|  | all6sites_DEDEED | 0.00353393 | 0.00011433 | 0.06 | 0.09 |
|  | all6sites_DEDEEE | 0.000454208 | 2.76E-06 | 0.01 | 0.00 |
|  | all6sites_DEEDDD | 0.000385243 | 3.07E-05 | 0.01 | 0.02 |
|  | all6sites_DEEDDE | 0.013654816 | 0.00028843 | 0.24 | 0.23 |
|  | all6sites_DEEDED | 0.007255316 | 0.000348577 | 0.13 | 0.28 |
|  | all6sites_DEEDEE | 0.006908702 | 0.000168436 | 0.12 | 0.14 |
|  | all6sites_DEEEDD | 0 | 6.04E-05 | 0.00 | 0.05 |
|  | all6sites_DEEEDE | 0.000254111 | 2.36E-05 | 0.00 | 0.02 |
|  | all6sites_DEEEED | 0.001962098 | 0.000229826 | 0.03 | 0.19 |
|  | all6sites_DEEEEE | 0.000312901 | 6.92E-05 | 0.01 | 0.06 |
|  | all6sites_EDDDDD | 0.012386591 | 0 | 0.21 | 0.00 |
|  | all6sites_EDDDDE | 0.022099972 | 0 | 0.38 | 0.00 |
|  | all6sites_EDDDED | 0.010322792 | 0 | 0.18 | 0.00 |
|  | all6sites_EDDDEE | 0.010879356 | 2.03E-06 | 0.19 | 0.00 |
|  | all6sites_EDDEDD | 0.001542308 | 0 | 0.03 | 0.00 |
|  | all6sites_EDDEDE | 0.001049624 | 5.33E-07 | 0.02 | 0.00 |
|  | all6sites_EDDEED | 0.002740457 | 0 | 0.05 | 0.00 |
|  | all6sites_EDDEEE | 0.02998212 | 7.07E-05 | 0.52 | 0.06 |
|  | all6sites_EDEDDD | 0.001530072 | 0 | 0.03 | 0.00 |
|  | all6sites_EDEDDE | 0.009304684 | 2.03E-05 | 0.16 | 0.02 |
|  | all6sites_EDEDED | 0.002081211 | 1.02E-05 | 0.04 | 0.01 |
|  | all6sites_EDEDEE | 0.05912259 | 0.00119145 | 1.02 | 0.96 |
|  | all6sites_EDEEDD | 0.007764484 | 0 | 0.13 | 0.00 |
|  | all6sites_EDEEDE | 0.022385526 | 0.000268003 | 0.39 | 0.22 |
|  | all6sites_EDEEED | 0.002371444 | 0 | 0.04 | 0.00 |
|  | all6sites_EDEEEE | 0.044149324 | 0.002634444 | 0.76 | 2.13 |
|  | all6sites_EEDDDD | 0.000394285 | 0 | 0.01 | 0.00 |
|  | all6sites_EEDDDE | 0.003599976 | 7.26E-05 | 0.06 | 0.06 |
|  | all6sites_EEDDED | 0.00511593 | 6.05E-05 | 0.09 | 0.05 |
|  | all6sites_EEDDEE | 0.02188693 | 0.000683594 | 0.38 | 0.55 |
|  | all6sites_EEDEDD | 0.000671327 | 3.78E-05 | 0.01 | 0.03 |
|  | all6sites_EEDEDE | 0.011474442 | 0.000398599 | 0.20 | 0.32 |
|  | all6sites_EEDEED | 0.00317418 | 0 | 0.05 | 0.00 |
|  | all6sites_EEDEEE | 0.02610455 | 0.001361755 | 0.45 | 1.10 |
|  | all6sites_EEEDDD | 0 | 1.13E-05 | 0.00 | 0.01 |
|  | all6sites_EEEDDE | 0.003557645 | 0.00014284 | 0.06 | 0.12 |
|  | all6sites_EEEDED | 3.65E-05 | 1.32E-05 | 0.00 | 0.01 |
|  | all6sites_EEEDEE | 0.042464152 | 0.000403209 | 0.73 | 0.33 |
|  | all6sites_EEEEDD | 0.00038038 | 7.54E-06 | 0.01 | 0.01 |
|  | all6sites_EEEEDE | 0.009927394 | 0.00034243 | 0.17 | 0.28 |
|  | all6sites_EEEEED | 0.002338171 | 1.26E-05 | 0.04 | 0.01 |
|  | all6sites_EEEEEE | 0.022095842 | 0.000790612 | 0.38 | 0.64 |
