# Supplementary material for: Uncovering Cis-Regulatory Elements Important for A-to-I RNA Editing in Fusarium graminearum
Source: mBio. 2022 Sep 14;13(5):e01872-22. doi: 10.1128/mbio.01872-22 (PMC9600606; doi:10.1128/mbio.01872-22)
Supplement: TABLE S4 [file mbio.01872-22-s0009.docx]

**Table S4** **Summary of primers used in this study.**

| Category | Primer name | Primer sequence (5’-3’)* |
| --- | --- | --- |
| Gene deletion | 330-1F | CTTGGATCAGGTAAATATACTCCGC |
|  | 330-2R | AATGCTCCTTCAATATCATCTTCTGTGAGAAAGTGGGCGATACAAGA |
|  | 330-3F | CGTCCGCAATGTGTTATTAAGTCGACCTTGTGAACCACTCGGGGATT |
|  | 330-4R | GAACTCGTTTCCTGCGGTGAC |
|  | 330-5F | TGTTCACCTGTTCGGATTCAC |
|  | 330-6R | GCACGGTGAGACTTTTGAATG |
|  | 330-7F | TCCACAATAGACCAAACCCAGTG |
|  | 330-8R | TTTGAGAACCTCGTGAACTGG |
|  | HYG-F | GGCTTGGCTGGAGCTAGTGGAGGTCAA |
|  | HT-R | GTCGACTTAATAACACATTGCGGACGT |
|  | HT-matchF | TCGGTCAATACACTACATGGCGTG |
|  | HT-matchR | GGATGAGGGCCACGAACGCCAGCAC |
|  | HT-detectF | GCCGATCTTAGCCAGACGAG |
|  | HT-detectR | GCGGTCGATGTGTCTGTCCTC |
|  | HT-855F | GCTAACTGAAGTAGATGCCGACCG |
|  | HT-856R | GCTACTGCTACAAGTGGGGCTGAT |
| Single nucleotide mutagenesis | 330-1(-1)-MF | GATGTGCTTC**G**AGCAAAGTACAAGG |
|  | 330-1(-1)-MR | CCTTGTACTTTGCT**C**GAAGCACATC |
|  | 330-1(+2)-MF | GATGTGCTTCTAG**G**AAAGTACAAGG |
|  | 330-1(+2)-MR | CCTTGTACTTT**C**CTAGAAGCACATC |
|  | 330-2(-2)-MF | GTCC**G**TAGGAGTTGATACCCG |
|  | 330-2(-2)-MR | CGGGTATCAACTCCTA**C**GGAC |
|  | 330-2(-1)-MF | GTCCC**G**AGGAGTTGATACCCG |
|  | 330-2(-1)-MR | CGGGTATCAACTCCT**C**GGGAC |
|  | 330-2(+1)-MF | GTCCCTA**C**GAGTTGATACCCG |
|  | 330-2(+1)-MR | CGGGTATCAACTC**G**TAGGGAC |
|  | 330-2(+2)-MF | GTCCCTAG**A**AGTTGATACCCG |
|  | 330-2(+2)-MR | CGGGTATCAACT**T**CTAGGGAC |
|  | 330-2(+3)-MF | GTCCCTAGG**C**GTTGATACCCG |
|  | 330-2(+3)-MR | CGGGTATCAAC**G**CCTAGGGAC |
|  | 330-3(-1)-MF | CACCGTGCT**T**AGGAAGCTACG |
|  | 330-3(-1)-MR | CGTAGCTTCCT**A**AGCACGGTG |
|  | 330-4(-2)-MF | GAGGAG**G**TAAGGTCTGAAGCC |
|  | 330-4(-2)-MR | GGCTTCAGACCTTA**C**CTCCTC |
|  | 330-4(-1)-MF | GAGGAGC**G**AAGGTCTGAAGCC |
|  | 330-4(-1)-MR | GGCTTCAGACCTT**C**GCTCCTC |
|  | 330-4(+1)-MF | GAGGAGCTA**C**GGTCTGAAGCC |
|  | 330-4(+1)-MR | GGCTTCAGACC**G**TAGCTCCTC |
|  | 330-4(+2)-MF | GAGGAGCTAA**A**GTCTGAAGCC |
|  | 330-4(+2)-MR | GGCTTCAGAC**T**TTAGCTCCTC |
|  | 330-4(+3)-MF | GAGGAGCTAAG**C**TCTGAAGCC |
|  | 330-4(+3)-MR | GGCTTCAGA**G**CTTAGCTCCTC |
| Double nucleotides mutagenesis | 330-2(-2,+1)-MF | GTCC**G**TA**C**GAGTTGATACCCG |
|  | 330-2(-2,+1)-MR | CGGGTATCAACTC**G**TA**C**GGAC |
|  | 330-2(-2,+2)-MF | GTCC**G**TAG**A**AGTTGATACCCG |
|  | 330-2(-2+2)-MR | CGGGTATCAACT**T**CTA**C**GGAC |
|  | 330-2(-2,+3)-MF | GTCC**G**TAGG**C**GTTGATACCCG |
|  | 330-2(-2,+3)-MR | CGGGTATCAAC**G**CCTA**C**GGAC |
|  | 330-2(+1,+2)-MF | GTCCCTA**CA**AGTTGATACCCG |
|  | 330-2(+1,+2)-MR | CGGGTATCAACT**TG**TAGGGAC |
|  | 330-2(+1,+3)-MF | GTCCCTA**C**G**C**GTTGATACCCG |
|  | 330-2(+1,+3)-MR | CGGGTATCAAC**G**C**G**TAGGGAC |
|  | 330-2(+2,+3)-MF | GTCCCTAG**AC**GTTGATACCCG |
|  | 330-2(+2,+3)-MR | CGGGTATCAAC**GT**CTAGGGAC |
|  | 330-4(-2,+2)-MF | GAGGAG**G**TAA**A**GTCTGAAGCC |
|  | 330-4(-2+2)-MR | GGCTTCAGAC**T**TTA**C**CTCCTC |
|  | 330-4(-2,+3)-MF | GAGGAG**G**TAAG**C**TCTGAAGCC |
|  | 330-4(-2,+3)-MR | GGCTTCAGA**G**CTTA**C**CTCCTC |
|  | 330-4(+2,+3)-MF | GAGGAGCTAA**AC**TCTGAAGCC |
|  | 330-4(+2,+3)-MR | GGCTTCAGA**GT**TTAGCTCCTC |
| Secondary structure change | 330-SSC1-MF | CAAAGA**ACAT**CTTCTAGCAAAG |
|  | 330-SSC1-MR | CTTTGCTAGAAG**ATGT**TCTTTG |
|  | 330-SSC2-MF | GGAGTTG**C**TA**G**CCGAGGCC |
|  | 330-SSC2-MR | GGCCTCGG**C**TA**G**CAACTCC |
|  | 330-SSC3-MF | GTCTCAC**GCA**GCTGAGGAAG |
|  | 330-SSC3-MR | CTTCCTCAGC**TGC**GTGAGAC |
|  | 330-SSC4-MF | CGAATTAAAG**T**GGCGGAGGAGC |
|  | 330-SSC4-MR | GCTCCTCCGCC**A**CTTTAATTCG |
| 3’UTR deletion | 330-Δ3’UTR-MR | TCAAGAAGTTGAGCTGTGCTCCAC |
|  | 330-Δ3’UTR-MF | GTTCGTGGAGCACAGCTCAACTTCTTGAGGGGTTATGCTACGTTATCAATTGAC |
| Ectopic expression | 330-pΔ3’UTR-F | AGGGAACAAAAGCTGGGTACCATGGATTATCAGCAAGGCAAC |
|  | 330-pΔ3’UTR-R | GCCCTTGCTCACCATAAGCTTTCAAGAAGTTGAGCTGTGCTCCAC |
|  | 330-pWT-F | AGGGAACAAAAGCTGGGTACCATGGATTATCAGCAAGGCAAC |
|  | 330-pWT-R | GCCCTTGCTCACCATAAGCTTCATTCATGCAGGGACAATAGGGATATC |
|  | 330-p5’part-1F | AGGGAACAAAAGCTGGGTACCATGGATTATCAGCAAGGCAAC |
|  | 330-p5’part-2R | ATTGAGACGTGGTAAAGTGTGATG |
|  | 330-p5’part-3F | TTCATCACACTTTACCACGTCTCAATCCGTCCGTCAATCAAGAAGCTG |
|  | 330-p5’part-4R | GCCCTTGCTCACCATAAGCTTCTATCCTTCACTGCGACCATCTC |
| Sanger sequencing | 330-SF | CCGTCCGTCAATCAAGAAGCTG |
|  | 330-SF-1 | GTTTCGCTTGACCTTGCCTCAG |
|  | 330-SR | GCACGGTGAGACTTTTGAATG |
| qRT-PCR | P100-qRT-F | CGGTGTCCCTAGGAGTTGATA |
|  | P100-qRT-R | CCAGATGCGTATGACTCTTTCC |
|  | Actin-qRT-F | ATCCACGTCACCACTTTCAA |
|  | Actin-qRT-R | TGCTTGGAGATCCACATTTG |

*Mutagenized nucleotides are red colored.
